# The assessment of dietary carotenoid intake of the Cardio-Med FFQ using food records and biomarkers in an Australian cardiology cohort: a pilot validation

**DOI:** 10.1017/jns.2024.6

**Published:** 2024-04-11

**Authors:** Teagan Kucianski, Hannah L. Mayr, Audrey Tierney, Hassan Vally, Colleen J. Thomas, Leila Karimi, Lisa G. Wood, Catherine Itsiopoulos

**Affiliations:** 1 School of Allied Health, Human Services and Sport, Faculty of Science and Engineering, La Trobe University, Bundoora, Victoria, Australia; 2 Centre for Functioning and Health Research, Metro South Hospital and Health Service, Brisbane, Queensland, Australia; 3 Department of Nutrition and Dietetics, Princess Alexandra Hospital, Woolloongabba, Queensland, Australia; 4 Greater Brisbane Clinical School, Faculty of Medicine, The University of Queensland, St Lucia, Queensland, Australia; 5 School of Allied Health, Health Implementation Science and Technology Centre, Health Research Institute, University of Limerick, Limerick, Ireland; 6 Institute for Health Transformation, Deakin University, Melbourne, Victoria, Australia; 7 Department of Physiology, Anatomy and Microbiology, School of Agriculture, Biomedicine and Environment, La Trobe University, Bundoora, Victoria, Australia; 8 Centre for Cardiovascular Biology and Disease Research, School of Agriculture, Biomedicine and Environment, La Trobe University, Bundoora, Victoria, Australia; 9 Florey Institute of Neuroscience and Mental Health, Pre-Clinical Critical Care Unit, University of Melbourne, Melbourne, Victoria, Australia; 10 School of Health and Biomedical Sciences, Department of Psychology, RMIT University, Melbourne, Victoria, Australia; 11 School of Biomedical Science and Pharmacy, University of Newcastle, Callaghan, New South Wales, Australia; 12 School of Health and Biomedical Sciences, RMIT University, Melbourne, Australia

**Keywords:** Carotenoid biomarkers, Dietary intake assessment, Food Frequency Questionnaire, Validation

## Abstract

Dietary carotenoids are associated with lower risk of CHD. Assessment of dietary carotenoid intake using questionnaires can be susceptible to measurement error. Consequently, there is a need to validate data collected from FFQs which measure carotenoid intake. This study aimed to assess the performance of the Cardio-Med Survey Tool (CMST)-FFQ-version 2 (v2) as a measure of dietary carotenoid intake over 12-months against plasma carotenoids biomarkers and 7-Day Food Records (7DFR) in an Australian cardiology cohort. Dietary carotenoid intakes (β- and α-carotene, lycopene, β-cryptoxanthin and lutein/zeaxanthin) were assessed using the 105-item CMST-FFQ-v2 and compared to intakes measured by 7DFR and plasma carotenoid concentrations. Correlation coefficients were calculated between each dietary method, and validity coefficients (VCs) were calculated between each dietary method and theoretical true intake using the ‘methods of triads’. Thirty-nine participants aged 37–77 years with CHD participated in the cross-sectional study. The correlation between FFQ and plasma carotenoids were largest and significant for β-carotene (0.39, *p*=0.01), total carotenoids (0.37, *p*=0.02) and β-cryptoxanthin (0.33, *p*=0.04), with weakest correlations observed for α-carotene (0.21, *p*=0.21) and lycopene (0.21, *p*=0.21). The FFQ VCs were moderate (0.3–0.6) or larger for all measured carotenoids. The strongest were observed for total carotenoids (0.61) and β-carotene (0.59), while the weakest were observed for α-carotene (0.33) and lycopene (0.37). In conclusion, the CMST-FFQ-v2 measured dietary carotenoids intakes with moderate confidence for most carotenoids, however, there was less confidence in ability to measure α-carotene and lycopene intake, thus further research is warranted using a larger sample.

## Introduction

Oxidative stress and inflammation are risk factors associated with the development of a range of chronic diseases including Cardiovascular Disease (CVD).^(1,2)^ Diet can influence the risk of chronic disease development and modulate these risk factors. A dietary pattern known to favourably reduce oxidative stress and inflammation is the Mediterranean Diet (MedDiet).^(3)^ The MedDiet pattern is predominantly a plant-based diet promoting a frequent and large consumption of fruits, vegetables and other plant-based foods including legumes and wholegrains,^(4)^ which are a major source of vitamins, minerals and fibre.^(5)^ Plant-based foods also contain bioactive constituents such as carotenoids, with fruits and vegetables being a concentrated source.

Carotenoids are naturally occurring compounds that are found in plants. Humans are unable to synthesise carotenoids and they must be consumed from dietary sources.^(6)^ Carotenoids are associated with many health benefits and through their established mechanistic properties can reduce oxidative stress and inflammation.^(1,2)^ This has been associated with a reduction in the risk of chronic diseases which have underlying oxidative and inflammatory pathways in their aetiology, including Coronary Heart Disease (CHD)^(7)^; the most prevalent form of CVD.^(8)^ There are >600 carotenoids found within nature and foods.^(9)^ The six major dietary carotenoids detectable in plasma, and thus most extensively examined in validation studies, include: β-carotene, α-carotene, lycopene, β-cryptoxanthin, lutein and zeaxanthin.^(10,11)^

It is important that measurement of the diet can be completed accurately when assessing diet–disease associations.^(12)^ Dietary evaluation can be undertaken via multiple self-report assessment methods, for example, food record (FR), 24-h food recall and FFQ. FFQs are advantageous since they can estimate nutrient intakes over longer periods of time, are low cost and relatively easy to use. Despite their frequent use, the accuracy of dietary information collected by FFQs is imperfect. Systematic and/or random measurement error tends to overestimate consumption, which is a significant limitation.^(13)^

Validation techniques are employed to determine the accuracy of particular methods used to collect data, including questionnaires.^(13)^ During validation of a FFQ, a reference method (e.g. FR or 24-h food recall) is often used for comparison.^(12,14)^ It is important to note that such self-reported reference methods are themselves open to the same random and systematic errors as the FFQ, which may impact the validation process through the perpetuation of correlated errors.^(15)^ To overcome this limitation, biochemical markers (biomarkers) can be used as the reference method given they provide an objective measure and have errors that are independent to the dietary tool being validated.^(13,16)^ Previous reports describe a dose–response relationship existing between carotenoid intake and subsequent concentration in plasma, suggesting that carotenoid biomarkers are a reliable proxy for dietary carotenoid intake^(7,17)^ The FFQ validation process can be enhanced by utilisation of two reference methods, i.e., biomarkers and traditional dietary assessment measures (e.g. FRs) in a triangulation validation technique known as the ‘methods of triads’, which allows dietary measures to be correlated against a theoretically true intake through derivation of a validity coefficient (VC).^(17)^

There is a scarcity of Australian FFQs developed to assess carotenoid intake (and as an extension, adherence to the MedDiet pattern) and even fewer tools which have been validated in a cohort with CHD using biomarkers or the methods of triads process.^(18–22)^ In 2013, we developed the Cardio-Med Survey Tool (CMST) FFQ to measure dietary intake in a multi-ethnic Australian cardiology population with an ability to measure MedDiet adherence through inclusion of foods that are consistent with the MedDiet pattern. The CMST-FFQ was found to be a reliable tool for measuring macro- and micronutrient intake.^(23)^ This tool was modified (CMST-FFQ-version-2 (v2)) to enable an assessment of carotenoid intake through expansion of the range and types of fruits and vegetables included.

The aim of the present pilot study was to assess the validity of the CMST-FFQ-v2 for estimating dietary carotenoid intake over the preceding year. Validity was assessed by comparing the assessment of the consumption of these compounds against those measured by 7DFR and objectively measured biomarkers (plasma carotenoid levels) in an Australian cardiology cohort.

## Methods

### Study design

Data was obtained from participants at study entry (baseline) in the AUStralian MEDiterranean diet (AUSMED) Heart Trial pilot study.^(24)^ The AUSMED Heart Trial is a multi-centre, randomised control MedDiet intervention for secondary prevention of CHD in a multi-ethnic Australian population. The intervention lasted for 6 months with a 12-month follow-up. Inclusion criteria included those who were ≥18 years, had adequate English comprehension for reading and writing and had experienced at least one acute coronary syndrome: acute myocardial infarction (AMI), angina pectoris with evidence of CHD, coronary artery bypass graft or percutaneous coronary intervention.

### Ethical standards disclosure

This study was conducted according to the guidelines laid down in the Declaration of Helsinki and all procedures involving research participants were approved by The Northern Hospital ethics committee (HREC P02/13), St Vincent’s Hospital ethics committee (HREC-A 016/13) and La Trobe University ethics committee (FHEC 13/159). Written informed consent was obtained from all subjects. The study is also registered on the Australian New Zealand Clinical Trials Registry (ACTRN12616000156482).

### Participants

Participants were recruited from two major hospitals in Melbourne, Australia, including inpatient and outpatient cardiology settings. A total of 65 participants were enrolled in the baseline phase of the AUSMED pilot study between 2014 and 2016. To be included in the present validation study, participants were required to have completed the CMST-FFQ-v2, a 7-day FR (7DFR) and provided a blood sample. One participant did not complete both FFQ and 7DFR and five participants had inadequate blood sample volumes; thus 59 participants had complete data across all three measurement methods. No participants were excluded based upon the percentage of questions omitted on the FFQ (cut-off for exclusion was <90% complete,^(18)^ however, under-reporters (*n* 15) and over-reporters (*n* 5) of energy intake determined by the Goldberg method (reported by Black^(25)^) were excluded from analysis. Under-reporters were defined as EI (energy intake):EER (estimated energy required) <0.75, normal reporters were defined as EI:EER ≥0.75–1.25 and over reporters as EI:EE >1.25. A final total of 39 participants were included in the validation analysis.

### Dietary intake

#### Food-frequency questionnaire

Dietary intake was assessed using the self-report semi-quantitative CMST-FFQ-v2, a paper-based modified version of the original 97-item CMST-FFQ, where design and validation has been previously described.^(23)^ The CMST-FFQ was originally developed to enable dietary assessment in a cardiology population and measure MedDiet adherence in Australia. Relevant modifications to the CMST-FFQ included the addition of several fruit categories (citrus, berries, melon, other, stone and dried), the red/orange vegetable category and two cereal categories (crispbreads/crackers and other grains). Fruits, vegetables and grains are key components of the MedDiet and concentrated sources of carotenoids, thus evaluating their consumption is crucial when assessing carotenoid intake. The CMST-FFQ-v2 consists of 105 items including a 51-item FFQ (of which 6 are specific to fruits and 11 to vegetable and legume intake), and 54 supplementary dietary questions: 14 portion questions, 30 diet questions and 10 food habit questions. The FFQ required participants to report their consumption of food/beverages over the preceding 12 months and provides a choice of 10 response categories ranging from ‘never’ up to ‘3 times per day’. Portion size photographs were used to provide estimates of food portions for 14 commonly consumed foods. Foods with no portion options were assigned median portions from the 2011/12 Australian National Nutrition survey,^(26)^ natural portion sizes, or as a last resort, portions recommended by the Australian dietary guidelines.^(27)^ The supplementary dietary questions encompassed information regarding fat and oil consumption, types of foods consumed, cooking methods, beverages and alcohol intake. Carotenoid bioavailability is subject to considerable variability, influenced by an array of factors both physiological and dietary. Carotenoids are lipophilic and demonstrate an increased bioavailability alongside the ingestion of dietary fats^(7)^. How carotenoids are consumed is important to consider, particularly in the context of the MedDiet, as carotenoid containing vegetables are often consumed alongside healthy fats like olive oil. The presence of these fats play a role in enhancing the absorption of carotenoids and this synergistic interaction is important in maximising the bioavailability of these crucial nutrients.

Demographic data, anthropometric data, past medical history, supplement usage and smoking history was also collected from participants in the self-reported health and lifestyle section of the CMST, at baseline study visits or from medical records.

#### Food records

Participants completed a 7DFR with details described in Mayr et al.^(28)^ Briefly, verbal and written instructions were provided regarding accurate completion prior to the baseline appointment by a research dietitian. Instructions included direction to record food and beverage information at the time of consumption, such as: amount/volume of all items, food type, brand, method of preparation and recipes. Food scales were advised to be used where possible, and where not possible, direction was given to use household measures. For meals not eaten at home, participants were asked to provide as much detail as possible with approximate amounts consumed using the tools provided in the written information.

Participants were instructed to complete the CMST-FFQ-v2 and 7DFR in the week prior to blood collection at the baseline appointment. All documents were checked for completeness by the study dietitian and nuances/missing information clarified with participants.

### Nutritional analysis

#### Food records

Dietary intake of carotenoids (β-carotene, α-carotene, lycopene, β-cryptoxanthin, lutein and zeaxanthin) from the 7DFR were calculated using the United States Department of Agriculture National Nutrient Database for Standard Reference (SR) Release 28 (USDA-SR-28)^(29)^ embedded within an Australian nutrient composition software program, FoodWorks (Version 10, Xyris Software Pty Ltd, Brisbane, Australia). Energy intake was assessed using the NUTTAB/AUSNUT databases within FoodWorks. The data was transposed from the 7DFR manually into FoodWorks by a study dietitian. For consistency of food items entry into FoodWorks, a food/product item repository was constructed to ensure identical selection of food items within the USDA-SR-28 database. The 7DFR analysis was also cross-checked by a dietitian to ensure consistency and accuracy.

#### Food-frequency questionnaire

Dietary intake of carotenoids from the FFQ within CMST-FFQ-2 (here on referred to as FFQ) was computed via a 3-step method:

1. Grams of food per day was computed by multiplication of frequency by portion size in grams.
2. A specifically constructed nutrient database utilising the USDA and NUTTAB/AUSNUT databases in FoodWorks contained the energy and carotenoid profile per gram for each food/beverage item in the FFQ. Each item in this database was multiplied by portion size intake (grams) per day. FFQ items that contributed to carotenoid intake (no matter how small) included: fruits, vegetables, processed meat, offal, cereals and grains (breakfast cereal, pasta, noodles, bread, crispbreads), dairy (yoghurt, cheese, milk), eggs, nuts and seeds, snacks (all except muesli bars and lollies), chocolate (milk and dark variety), meals not prepared at home (all items), herbs and spices (oregano, curry powder, cinnamon, chilli), condiments (lemon juice, tomato sauce, pepper), margarine and butter, nut spreads, mayonnaise and salad dressings, and beverages (herbal tea, fruit juice, red wine and cider).
3. Total daily carotenoid intake was obtained by tallying daily individual carotenoid intake across each food/beverage item consumed.

#### Plasma carotenoid biomarkers

Fasting blood samples were collected by experienced personnel using standard venepuncture techniques. Upon collection, blood samples were processed immediately and centrifuged, with plasma collected and stored in aliquots at –80°C until analysis. The tubes containing plasma samples to be analysed for carotenoids were immediately wrapped in foil to minimise light exposure. Plasma carotenoid samples were sent to an external laboratory (University of Newcastle, Newcastle, NSW, Australia) for analysis. High Performance liquid chromatography methodology was used to determine β-carotene, α-carotene, β-cryptoxanthin, lycopene and lutein/zeaxanthin (combined) concentrations in plasma. Total carotenoid concentration was calculated from the addition of all measured plasma carotenoids. All extractions were carried out under red light in a darkened laboratory, using validated methodology as described in Wood et al.^(30)^ Sample carotenoid peaks were identified and quantified using Agilent 1200 Series High Performance Liquid Chromatograph with Chemstations software (Agilent Corporation, Germany).^(30)^ Separately, serum cholesterol was measured at a commercial laboratory (Dorevitch Pathology Pty Ltd, Heidelberg, VIC, Australia) using an automated blood analyser (ADVIA 2400 Chemistry System, Siemens).

### Statistics

Descriptive statistics for baseline characteristics were presented as means ± standard deviation (SD), medians (interquartile range (IQR)) or frequencies (percentage) as appropriate. Carotenoid intakes measured from the FFQ and 7DFR were adjusted for energy intake using the nutrient residual method.^(31)^ Differences between measured intakes from the two dietary methods were examined using Wilcoxon-signed rank-test or independent Student’s *t*-test. Plasma carotenoid biomarker concentrations were adjusted for plasma cholesterol concentrations using the residual method^(18)^ due to a relationship existing between serum cholesterol and carotenoid levels.^(18)^

Spearman’s Rho ( 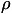 ) or Pearson correlation (*r*) coefficients were used as measures of correlation to assess the validity between the three dietary assessment methods (FFQ vs. 7DFR, FFQ vs. biomarker and 7DFR vs. biomarker) for each individual carotenoid and total carotenoid intake, depending on variable distribution. Correlations were evaluated as poor (<0·2), moderate (0·2–0·6) or good (>0·6).^(18,32)^ Correlations between known confounding variables (including body mass index (BMI), gender, age, supplement use and smoking history)^(19,22,33)^ and measured carotenoid intakes from the FFQ and 7DFR were assessed using Spearman correlation (ρ) coefficients to determine need for partial correlations (refer to Supplementary Materials 2, Table S1 and S2). No significant correlations were observed, thus obviating the need for partial correlations.

Correlations between each of the dietary methods were utilised to enable calculation of the VC between theoretical true intake and estimated intakes from FFQ, 7DFR (the reference method) and plasma carotenoid biomarkers using the methods of triads.^(16,34)^ Once correlation coefficients had been estimated, the following equations were utilised to calculate the VC ( 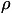 ) for each carotenoid measurement method with 95% CI:
(1)

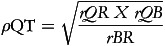



(2)

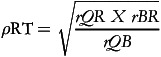



(3)

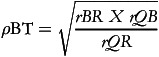




where *T* = true unknown long-term dietary intake, *r* = correlation coefficient; *Q* = FFQ, *R* = 7DFR; *B* = biomarker. This analysis assumes random errors in each of the methods are uncorrelated and a positive linear correlation exists between estimations of true intake and dietary intake.^(33,35)^ Ocke and Kaaks^(16)^ suggests that the range for the VC utilises the estimated VC as the upper limit for all measures. The correlation coefficient between FFQ and biomarker is used as the lower limit for both FFQ and biomarker and correlation coefficient between 7DFR and biomarker is utilised as the lower limit for the 7DFR.^(18,32)^ VCs were classified as weak (*ρ* < 0.2), moderate (0.2 ≤ *ρ* ≤ 0.6) and high (*ρ* > 0.6).^(16,36)^

Analyses were performed using the statistical software SPSS® version 27 (IBM Corp, released 2021) with reported *p*-values being two-tailed and the level of significance level set at 5%.

## Results

Demographic and clinical characteristics are presented in Table 1. The mean age of participants was 63.5 years, and a large proportion (87.2%) were male. The mean BMI of participants was 29.1 kg/m^2^, 17.9% were current smokers and 74.4 % of the cohort had experienced an AMI.

**Table 1.** Characteristics of study participants (*n* 39)

Variable	*n*	%
Gender		
Male	34.0	87.2
Female	5.0	12.8
Age (years)*	63.5	9.7
BMI (kg/m^2^)*	29.1	4.8
Smoking status		
Current	7.0	17.9
Non-smoker	32.0	82.1
Supplement use		
None	17.0	53.8
Any supplement	18.0	46.2
Multi-vitamin†	4.0	10.3
Medical history		
No AMI	10.0	25.6
AMI	29.0	74.4

SD, standard deviation; BMI, body mass index; AMI, acute myocardial infarction.

* Mean values and standard deviation.

† Only 2 of 4 participants documented brand of multivitamin consumed with neither documented brand containing carotenoids.

Table 2 presents the crude and energy-adjusted carotenoids as measured by the FFQ and 7DFR. The mean energy intake measured by the FFQ was less than the 7DFR although not significantly different. The median intake of both crude and energy-adjusted β-carotene, α-carotene, lycopene and total carotenoids was lower in the FFQ compared to the 7DFR with all differences statistically significant. Intakes determined by the FFQ ranged from 1.08-fold lower for total carotenoid intake to greater than 3-fold lower for α-carotene intake for both crude and energy-adjusted measures. The median FFQ intake for crude lutein/zeaxanthin was over 2-fold higher than estimated by the 7DFR (3588.5 (2021.2–6031.9) vs. 1667.3 (1239.7–3588.6) µg/d, *p*=0.004), with the same trend identified for energy-adjusted values (3813.8 (1267.5–3656.6) vs. 1877.1 (1267.5–3656.6) µg/d, *p*=0.002).

**Table 2.** Crude and energy-adjusted carotenoid intake measured from the FFQ and 7DFR (*n* 39)

	FFQ (µg/d)		7DFR (µg/d)		
Nutrient	Median	IQR	Median	IQR	*p*-value*
Crude					
Energy (kJ)	8408.1	7576.5–10068.9	7966.0	6335.6–9673.0	0.078^†^
β-carotene	2954.9	2246.8–4691.1	3865.8	2692.7–5378.6	0.032
α-carotene	298.6	172.1–448.6	974.2	473.2–1536.9	<0.001
β-cryptoxanthin	166.2	116.9–222.0	117.7	37.2–238.3	0.577
Lycopene	1705.6	1158.0–2284.2	2369.8	1255.3–5905.2	0.001
Lutein/zeaxanthin	3588.5	2021.2–6031.9	1667.3	1239.7–3588.6	0.004
Total carotenoids	8675.7	6035.4–13187.4	9349.3	7306.8–20024.0	0.042
Energy adjusted^‡^					
β-carotene	3280.7	1984.7–4357.2	4043.1	2595.6–5970.3	0.012
α-carotene	298.9	587.4–1590.8	973.5	587.4–1590.8	<0.001
β-cryptoxanthin	159.0	46.4–253.7	115.7	46.4–253.7	0.635
Lycopene	1733.8	1346.1–5879.6	2332.9	1346.1–5879.6	0.002
Lutein/zeaxanthin	3813.8	1267.5–3656.6	1877.1	1267.5–3656.6	0.002
Total carotenoids	9285.4	7083.0–20454.1	9635.4	7083.0–20454.1	0.032

FFQ, food frequency questionnaire; IQR, interquartile range; 7DFR, 7-day food record.

* *p*-value examining differences between FFQ and 7DFR measured intakes using Wilcoxon-signed rank test.

† Independent Student’s *t*-test *p*-value presented.

‡ Daily carotenoid intake measured by FFQ and 7DFR was adjusted for daily energy intake.

Table 3 presents the crude and cholesterol-adjusted median and IQR of plasma biomarker measurements for each of the five carotenoids, total carotenoids and cholesterol. Cholesterol adjusted median plasma biomarker concentrations ranged from 0.04 mg/l (α-carotene) to 1.30 mg/l (total carotenoid), with crude values remaining almost identical to cholesterol adjusted values (Table 3).

**Table 3.** Crude and cholesterol-adjusted plasma carotenoid measures (*n* 39)

	Crude plasma biomarker (mg/l)		Cholesterol-adjusted plasma biomarker (mg/l)^*^	
Carotenoid	Median	IQR	Median	IQR
β-carotene	0.46	0.23–0.80	0.47	0.21–0.81
α-carotene	0.04	0.02–0.06	0.04	0.02–0.06
β-cryptoxanthin	0.15	0.09–0.21	0.15	0.09–0.21
Lycopene	0.16	0.12–0.25	0.16	0.11–0.25
Lutein/zeaxanthin	0.34	0.24–0.46	0.35	0.23–0.46
Total carotenoids	1.30	0.83–1.89	1.30	0.84–1.90
Total cholesterol	3.60	2.90–3.90	–	

IQR, interquartile range.

* Plasma biomarker carotenoid levels were adjusted for plasma cholesterol concentration.

Table 4 presents the Spearman correlation coefficients between all the measured carotenoid values from the dietary assessment methods (FFQ and 7DFR) and the plasma biomarkers. Moderate correlations between the energy-adjusted carotenoids measured by FFQ and 7DFR were observed for all carotenoids except for lycopene. The strongest and statistically significant correlations were observed for β-carotene and lutein/zeaxanthin (ρ=0.39 and 0.32, *p*<0.05, respectively). All other carotenoids had non-significant correlations with the poorest correlation observed for lycopene (ρ=0.15, *p*>0.05). The crude correlations remained similar with a trend towards some smaller correlations compared to the energy-adjusted values (except for lycopene which increased marginally in correlation strength from 0.15 to 0.22, a difference of 0.07).

**Table 4.** Spearman’s correlations coefficients (ρ) for crude and energy-adjusted carotenoids measured by FFQ, 7DFR and biomarkers (*n* 39)

Nutrient/variable	7DFR-FFQ		FFQ-biomarker		7DFR-biomarker	
	ρ	*p*-value	ρ	*p*-value	ρ	*p*-value
Crude						
β-carotene	0.39	0.015	0.39	0.013	0.52	<0.001
α-carotene	0.19	0.25	0.15	0.36	0.47	0.003
β-cryptoxanthin	0.22	0.17	0.34	0.035	0.47	0.003
Lycopene	0.22	0.19	0.19	0.25	0.26	0.11
Lutein/zeaxanthin	0.33	0.042	0.28	0.09	0.34	0.036
Total carotenoids	0.16	0.34	0.37	0.022	0.27	0.10
Energy adjusted						
β-carotene	0.39	0.013	0.39	0.014	0.44	0.005
α-carotene	0.21	0.21	0.21*	0.21	0.39	0.014
β-cryptoxanthin	0.24	0.15	0.33	0.039	0.45	0.004
Lycopene	0.15	0.36	0.21	0.21	0.23	0.17
Lutein/zeaxanthin	0.32	0.049	0.25^*^	0.13	0.29	0.07
Total carotenoids	0.25	0.13	0.37	0.019	0.25	0.13

FFQ, food frequency questionnaire; 7DFR, 7-d food record.

ρ, Spearman’s correlations.

* Pearson correlation presented.

Moderate correlations were observed for all energy-adjusted carotenoids measured by FFQ and biomarker, while significant correlations observed for β-carotene, β-cryptoxanthin and total carotenoids with the strongest correlations observed for β-carotene and total carotenoids ( 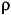 =0.39 and 0.37, *p*<0.05, respectively). The remaining carotenoids demonstrated non-significant correlations with the poorest correlations observed for α-carotene ( 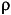 =0.21, *p*>0.05) and lycopene ( 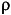 =0.21, *p*>0.05). The crude correlations for the FFQ vs. biomarker remained static or trended towards being marginally smaller compared to energy-adjusted values (with lutein the only carotenoid to marginally increase). Crude and energy-adjusted correlations tended to be stronger between the biomarker and 7DFR compared to the biomarker and FFQ, except for total carotenoids.

The correlations between each of the three measurement methods (FFQ, 7DFR and biomarkers) for each measured carotenoid were used to calculate the VCs using the methods of triads. Table 5 presents these calculated VCs alongside the 95% CI and the range for the VC. The energy-adjusted VCs for the FFQ (against true intake) for all measured carotenoids were moderate except for total carotenoids which were classified as high. VCs for the FFQ ranged from 0.33 (α-carotene) to 0.61 (total carotenoids). The FFQ VCs for total carotenoids and β-carotene were the strongest ( 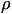 =0.61 and 0.59 respectively), followed by lutein/zeaxanthin, β-cryptoxanthin and lycopene ( 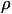 =0.52, 0.42 and 0.37, respectively), with α-carotene displaying the poorest VC ( 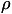 =0.33). The FFQ VCs were generally smaller in comparison to the 7DFR and biomarker VCs; the exception being for lutein/zeaxanthin which was stronger than the VC for biomarkers and total carotenoids which was larger than the VC for 7DFRs. All trends observed remained similar for crude VCs, although a trend towards larger VCs were observed for most carotenoids.

**Table 5.** Validity coefficient presented for FFQ, 7DFR and biomarker calculated using methods of triads (*n* 39)

Carotenoid	FFQ			7DFR			Biomarker			Range for the validity coefficient		
	*ρ*QT	95% CI		*ρ*RT	95% CI		*ρ*BT	95% CI		FFQ	7DFR	Biomarker
Crude												
B-carotene	0.55	0.28	0.74	0.71	0.51	0.84	0.72	0.52	0.84	0.39–0.55	0.52–0.71	0.39–0.72
A-carotene	0.25	−0.07	0.52	0.76	0.58	0.87	0.61	0.36	0.78	0.15–0.25	0.47–0.76	0.15–0.61
B-cryptoxanthin	0.40	0.10	0.64	0.56	0.30	0.74	0.84	0.71	0.91	0.34–0.40	0.47–0.56	0.34–0.84
Lycopene	0.39	0.08	0.63	0.55	0.28	0.74	0.48	0.19	0.69	0.19–0.39	0.26–0.55	0.19–0.48
Lutein/zeaxanthin	0.52	0.24	0.72	0.63	0.39	0.79	0.53	0.26	0.72	0.28–0.52	0.34–0.63	0.28–0.53
Total carotenoids	0.47	0.18	0.68	0.34	0.03	0.59	0.79	0.63	0.88	0.37–0.47	0.27–0.34	0.37–0.79
Energy adjusted												
B-carotene	0.59	0.34	0.76	0.66	0.44	0.81	0.66	0.44	0.81	0.39–0.59	0.44–0.66	0.39–0.66
A-carotene	0.33	0.02	0.58	0.63	0.39	0.79	0.62	0.38	0.78	0.21–0.33	0.39–0.63	0.21–0.62
B-cryptoxanthin	0.42	0.12	0.65	0.56	0.30	0.74	0.79	0.63	0.88	0.33–0.42	0.45–0.56	0.33–0.79
Lycopene	0.37	0.06	0.61	0.41	0.11	0.64	0.55	0.28	0.74	0.21–0.37	0.23–0.41	0.21–0.55
Lutein/zeaxanthin	0.52	0.24	0.72	0.62	0.38	0.78	0.48	0.19	0.69	0.25–0.52	0.29–0.62	0.25–0.48
Total carotenoids	0.61	0.36	0.78	0.40	0.10	0.64	0.62	0.38	0.78	0.37–0.61	0.25–0.40	0.37–0.62

FFQ, food frequency questionnaire; 7DFR, 7-d food record.

*ρ*QT, validity coefficient of the questionnaire; *ρ*BT, validity coefficient of the biomarker; *ρ*RT, validity coefficient for the 7DFR.

The lower limit is ρ FFQ-biomarker for the FFQ and the biomarker and ρ 7DFR-biomarker for the 7DFR, and the upper limit is calculated by the method of triads.

## Discussion

The CMST-FFQ-v2 was developed to measure diet quality and adherence to traditional dietary patterns, such as the Mediterranean diet, in a culturally diverse Australian cardiology population. We previously demonstrated that the FFQ has good test–retest reliability and moderate validity against 7DFR in measuring energy, protein, carbohydrate and selected micronutrient intakes.^(23)^ The aim of this current study was to compare the CMST-FFQ-v2 in measuring the energy-adjusted dietary carotenoid intake with intake estimated from a 7DFR and from plasma carotenoid concentrations, in a cohort of individuals with CHD. This assessment of the validity of the FFQ involved the calculation of correlation coefficients and VCs. The results demonstrated a moderate and significant correlation between the FFQ and plasma biomarker for β-carotene, β-cryptoxanthin and total carotenoids, while the FFQ VCs demonstrated a moderate to strong correlation for all measured carotenoids.

Dietary carotenoid intakes were energy adjusted and analysed both by FFQ and 7DFR. The mean dietary carotenoid intakes measured by the FFQ were within the ranges observed in several other studies except for α-carotene and lycopene, which were lower in our study.^(18–21,37–39)^ This may indicate that our FFQ is not sensitive enough to adequately capture intake of both α-carotene and lycopene, whereas it is comparable to other FFQs for the balance of carotenoids measured.

Weighed FRs are the gold standard in food intake methodology and usually the first method of choice when validating a FFQ.^(13)^ In this study we have used the 7DFR as the method of reference, and additionally, we used the objective measure of plasma carotenoids (biomarkers) as another method of comparison through application of the method of triads. Three out of the five FFQ-measured carotenoids (β-carotene, α-carotene, lycopene), plus total carotenoids, had significantly smaller mean intakes than those reported from the 7DFR. Typically, FFQs are recognised to overestimate energy and nutrient intake compared to other dietary assessment measures.^(10,32)^ Our observations may be explained by the allocation of median serving size when portion selection was unavailable. This occurred for the red/orange vegetables group, which are indicators of α-carotene and lycopene intake.^(11)^ Additionally, aggregating individual foods into a single food group may cause dilution of true measured intake,^(13)^ e.g. α-carotene rich foods (orange/yellow vegetables and fruits) and lycopene rich foods (tomato and watermelon)^(11)^ are combined together or with other foods that differ in carotenoid composition and concentration.^(13)^ This can also be problematic when the individual foods within a composite group are not consumed in the same frequency or portion^(13)^ leading to a reduced ability to differentiate between single food items.

Plasma carotenoids have been shown to be a useful and objective biomarker for fruit and vegetable intakes, which are the main food sources of carotenoids,^(10,17)^ and a reliable method for prediction of dietary carotenoid intake.^(10,21)^ Plasma carotenoid concentration can however be impacted by external factors outside of dietary intake, for example: baseline plasma carotenoid concentration of an individual,^(10,21)^ physiological variability in absorption and digestion, genetic and lifestyle factors (e.g. gender, age, BMI, smoking history),^(12,13)^ cooking methods, amount of fat consumed in meals (as carotenoids are fat soluble) and individual vitamin A status.^(10)^ As a result of the random variability influencing plasma concentrations unrelated to dietary intake, correlation coefficients observed between FFQ intake and biomarkers are often less than 0.4,^(17,18,40)^ as was the case in our study.

There is a high degree of variability of reported correlations for dietary intake and plasma concentrations among different studies. A review by Burrows et al.^(10)^ incorporating 124 international studies identified correlations between FFQ intake and carotenoid biomarkers ranging from 0.26 to 0.39. This is comparable to the correlation range observed in our study (0.21–0.39). Individual carotenoid correlations observed in the review by Burrows et al.^(12)^, and our study were also similar, except for β-carotene, where we identified a larger correlation (0.39 vs. 0.27) and α-carotene, where we recorded a smaller correlation (0.21 vs. 0.34). Correlations observed in our study for β-cryptoxanthin (ρ=0.33), lutein/zeaxanthin (ρ=0.25), and lycopene (ρ=0.21), were within the range reported in three Australian validation studies: β-cryptoxanthin, -0.002–0.46; lycopene, 0.13–0.29; lutein/zeaxanthin, 0.03–0.29.^(18–20)^ The correlations in our study were observed to be larger for β-carotene (0.39 vs. 0.22–0.28) compared to the Australian studies while marginally lower for α-carotene (0.21 vs. 0.26–0.36).

Carotenoids that are ubiquitous in the food supply and those consumed in larger quantities showed stronger correlations between dietary intake and plasma level, for example β-carotene. Additionally, β-carotene is not closely regulated by a homeostatic mechanism (like some other carotenoids),^(14)^ making its plasma concentration more reflective of dietary intake. Despite α-carotene being abundant in the diet (like β-carotene), poorer correlations were observed. This may be attributable to various influencing factors. Firstly, the mixed food groupings described earlier may have diluted true intake. Secondly, food preparation and cooking techniques that impact α-carotene bioavailability^(11)^ may not have been captured adequately. Lastly, the portion size of the α-carotene rich vegetable food group (i.e. orange/red vegetables) was the only main vegetable class determined by assigning a median value for portion size rather than by self-selection. The literature reports that when subjects can select their portion size, correlation coefficients are typically higher.^(13)^

The methods of triads is a mathematical triangulation approach using comparisons between three different and independent measures of the variable being assessed to estimate a VC between each measurement method and the subjects’ estimated true habitual intake.^(32–34,40)^ This technique assumes that any errors associated with each method are independent of each other.^(32)^ The VCs for each carotenoid measured were larger than their respective correlation coefficients, suggesting that the triads method (utilising both FFQ and 7DFR data) is a more predictive technique for determining serum carotenoid concentrations than using a single dietary assessment method.^(41)^ Artificially high VCs may result from differences in assessment of carotenoid intake time frames, i.e. the FFQ and 7DFR being completed the week prior to plasma carotenoid (biomarker) collection. In our study the observed FFQ VCs of measured carotenoids were all moderate-to-high (ranging from 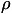 =0.33–0.61) suggesting the FFQ is a relatively reliable tool for measuring carotenoid intake^(38)^. The FFQ VCs of carotenoids vary considerably between studies, with many only presenting VCs for β-carotene,^(16,35,40,42–44)^ thus making comparisons difficult. For the limited studies that examined the same five carotenoids as our study, the observed VCs were wide and ranged from 0.19 to 0.84 in an Australian study,^(18)^ and 0.31 to 0.98 in two studies from the Americas.^(38,39)^ The VCs observed in our study were similar or smaller, which may be attributable to the differences in sample sizes, populations examined and cultural food preferences.

As previously noted, 7DFR were used as a surrogate measurement for the gold standard weighed FR. The 7DFR VCs for all carotenoids, with exception of lutein/zeaxanthin, were stronger compared to the FFQ VCs. Similar trends have been observed for individual carotenoids in some studies,^(16,35,39)^ while others have highlighted a contrary position.^(18,38,40)^ Stronger VCs are typically expected for FRs due to there being a greater level of accuracy in the capture of true foods consumed and cooking methods; and less potential for overestimation, as compared with FFQs.^(40)^ When FFQ VCs were compared to biomarker VCs, the majority were smaller, except for lutein/zeaxanthin and total carotenoids. This trend is different to what has been observed in studies which report on a range of carotenoids.^(16,18,38,40,45)^ While our results are not typical, similar findings have been observed to ours in studies that reported results based on a single carotenoid, for example, Daures et al.^(35)^ reported β-carotene VCs for FFQ and biomarker as 0.39 and 0.85, respectively, while Burri et al.^(41)^ reported lycopene VCs for the FFQ and biomarker of 0.49 and 0.66, respectively. Many of the inconsistencies observed between the results in validation studies and in comparison to our study may be consequential of differences between the studies; utilisation of different FFQs, time frames assessed by reference methods^(12,17,38)^ and biomarker concentration may vary if there are differences in laboratory testing and/or the isomers measured.^(39)^

Of particular importance is the difference in time frame assessed of carotenoid intake for each measurement method within the current study. The FFQ measured intake over the preceding 12-months, the FR measured intake over 7-d, while carotenoid biomarkers likely represent the previous weeks to months of carotenoid dietary exposure.^(46)^ When making comparisons, it is desirable that each method assesses intake over the same time frame,^(13)^ this is particularly important for carotenoids as their intake is subjected to wide seasonal variation. This mismatch of time frames in this study may reduce potential for detection of statistically significant relationships, reduce predictive performance and underestimate true correlations which may have been observed within our study.^(18)^ Increasing the length of the reference method through the application of multiple 7DFRs (i.e. collected every 3 months over a 12-month time frame) would allow a more comparable level of habitual intake to dietary information collected in the FFQ, and also improved capture of seasonal effects. Despite this time frame limitation, FFQs offer advantages over 7DFR and biomarkers; they are easier to use, have reduced participant burden, ability to pick up on seasonal variation and be utilised within large populations,^(13)^ making them beneficial measurement tools.

A key strength of the present study was the use of plasma biomarkers as an objective and independent measure of nutrient intake to validate FFQ estimated intakes^(12)^ and the use of the methods of triads which assists with correction of biases of correlated errors between dietary intake methods.^(16,38)^ This study is also one of very few which compares multiple dietary methods using a spectrum of carotenoids. Lastly, is the unique design of the FFQ which has a focus on the carotenoid-rich MedDiet pattern and assesses carotenoid-rich foods not often assessed by other FFQs (e.g. herbs and spices, condiments and mixed tomato containing dishes).

Several limitations of the present study should be noted. First, the relatively small sample size of participants (*n* 39) may have resulted in underpowering and difficulty in reliably detecting significant correlations. Other scientific literature suggests that a minimum desirable sample size for validation studies is between 50 (when using biomarkers)^(47)^ and 100 participants.^(13)^ In addition, females were under-represented in our sample, which limits the generalisability. This is a common issue in clinical trials of CHD.^(48)^ Second, the assessment of reproducibility was not investigated due to the nature of the data collection, which utilised baseline data from the AUSMED Heart Trial. Third, was the use of the USDA database, which is based on the US food supply and may not accurately reflect nutrient composition in the Australian food supply,^(21)^ and thus may have reduced the likelihood of detecting relationships. Fourth, there is debate whether a single blood measurement can reliably detect serum biomarker concentrations.^(18,40)^ due to individual variability and daily fluctuations.^(49)^ Fifth, relates to the order of completion of the FFQ and 7DFR. While the 7DFR and CMST were instructed to be completed 1 week prior to the study appointment, no instruction was provided regarding the order of completion. This is a potential limitation of the study as ideally the test instrument (i.e. FFQ) should be administered prior to the reference method (i.e. 7DFR) in order to prevent learned behaviours and biased responses.^(13)^ Last, mis-reporting of intake by participants using the FFQ can be impacted by social desirability bias or recall bias (memory) which can reduce accuracy of reported intake^(49)^ in comparison to objectively measured intakes (i.e. biomarkers).

Further research is warranted using increased sample size, assessment of reproducibility and exploring use of alternative biomarkers (including skin and adipose tissue), which may provide a more suitable prediction of longer-term dietary carotenoid intake compared to plasma carotenoids.^(50)^ Additionally, potential FFQ modifications to improve accuracy of dietary carotenoid measurement include: expansion of groupings of similar foods to individual foods (this must be balanced against the desired FFQ length), and separation of the orange/red vegetable food groups alongside provision of photograph portion references to enable selection of portion size.

In conclusion, this study demonstrated that the CMST-FFQ-v2 was able to estimate carotenoid intakes with moderate confidence for most of the measured carotenoids within this Australian cardiology cohort. Significant correlations observed between FFQ estimated intake of β-carotene, β-cryptoxanthin, and total carotenoid with plasma biomarkers and the moderate-strong FFQ VCs observed for all measured carotenoids. There was however less confidence in the FFQ’s ability to accurately measure intakes of α-carotene and lycopene due to the poorer correlations and VCs observed. Addressing limitations, making suggested future revisions for the FFQ and conducting a larger-scale investigation, may assist to strengthen the ability of the FFQ to accurately measure dietary carotenoid intake.

## Supporting information

Kucianski et al. supplementary material 1 Kucianski et al. supplementary material

Kucianski et al. supplementary material 2 Kucianski et al. supplementary material

## Supplementary material

The supplementary material for this article can be found at https://doi.org/10.1017/jns.2024.6.
