# Supplementary material for: The assessment of dietary carotenoid intake of the Cardio-Med FFQ using food records and biomarkers in an Australian cardiology cohort: a pilot validation
Source: J Nutr Sci. 2024 Apr 11;13:e20. doi: 10.1017/jns.2024.6 (PMC11016364; doi:10.1017/jns.2024.6)
Supplement: Kucianski et al. supplementary material 1 — Kucianski et al. supplementary material [file S2048679024000065sup001.docx]

**THE CARDIO-MED SURVEY**

**TOOL v2**


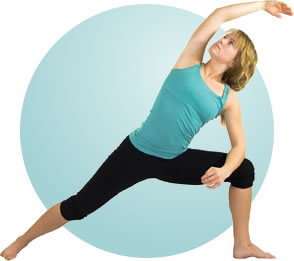

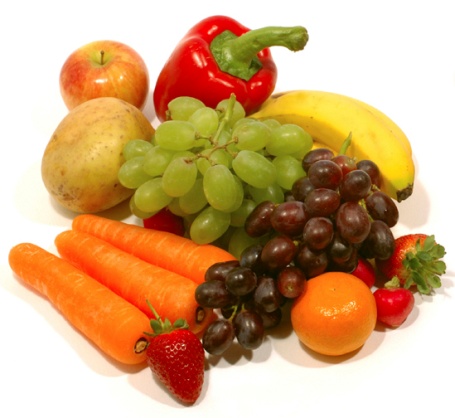
**[
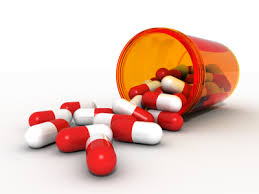
](http://www.google.com.au/imgres?q=medications&sa=X&biw=1536&bih=773&tbm=isch&tbnid=67GdTBFpuB7eiM:&imgrefurl=http://suneeldhand.com/2013/02/27/most-commonly-prescribed-medications/&docid=VwAff4wRb_OqnM&imgurl=http://suneeldhand.com/wp-content/uploads/2013/02/Medications.jpg&w=400&h=300&ei=993kUfq2K8nwlAWz94HIAg&zoom=1&iact=hc&vpx=2&vpy=374&dur=811&hovh=194&hovw=259&tx=131&ty=97&page=2&tbnh=156&tbnw=237&start=30&ndsp=39&ved=1t:429,r:61,s:0,i:274)**

**INSTRUCTIONS**

- The following questions ask about your lifestyle, background, medical history and diet that relate to your health.
- When answering questions please ***tick ONE box only unless otherwise specified***. Please mark your response as follows 🗹
- Please answer every question and ***do not leave any answer blank*** - however if you don’t feel comfortable answering any of these questions, please leave the answer blank.
- If you find that your answer will not fit the answer categories provided, please tick the closest answer OR the box named ‘other, please specify’ and briefly describe your answer.
- All the answers you provide will remain confidential.


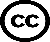
Kucianski *et al.*  2020

***The following questions will ask you about your lifestyle, background and medical history.***

**1. What is your postcode? _______________________**

**2.** **What is your gender?**

- Male
- Female

**3. What is your date of birth?** _ _ /_ _ / _ _ _ _

**4. What country were you born in?**

- Australia ***(Skip to Question 5)***
- Other please specify _____________________

**What year did you migrate to Australia?** ________________________________***(Skip to Question 6)***

**5. Are you of Aboriginal or Torres Strait Islander origin?** *(Tick ALL that apply)*

- No
- Yes Aboriginal
- Yes Torres Strait Islander

**6.** **What country were your parents born in?**

**Mother**

- Australia
- Other please specify______________________

**Father**

- Australia
- Other please specify ______________________

**7**. **Do you speak a language other than English at home?**

- No, English only
- Yes, please specify_________________________

**8. What is your marital status?**

- Married
- Not married but in a long term relationship.
- Widowed
- Separated
- Divorced
- Single

**9.** **Including yourself, how many people live in your household?**

- 1
- 2
- Other, please specify _______________________

**10. What is the highest level of education you have completed?**

- Never attended school
- Primary school
- Secondary school up to year 10
- Secondary school up to year 11/12 (or equivalent)
- Trade school / TAFE
- University

**11. Do you have a paid job at present?**

- No
- *Yes* ***(Skip to Question 13)***

**12. How would you describe yourself?**

- Homemaker
- Retired
- Unemployed
- Student
- Permanently unable to work / ill
- Other, please specify _______________________

**13**. **Which of the following categories best describes your current area of employment OR previous employment if not currently working?**

- **Managers** e.g. general manager, school principle, director of nursing, farm manager
- **Professionals** e.g. doctor, teacher, nurse, scientist, accountant
- **Associate professionals** e.g. police officer, technician, youth worker
- **Tradespersons and related workers** e.g. hairdresser, gardener, florist, mechanic, cook

***(Continue to next column)***

- **Clerical and services worker** e.g. receptionist, flight attendant, hospitality worker, sales assistant, child care worker, telemarketer
- **Intermediate production and transport workers** e.g. machine operator, bus driver
- **Labourers and related workers** e.g. cleaner, factory worker
- **I have never worked**

***Income is very important in understanding health, as it may influence the health services a person can access***

**14. Which number best describes your total household income before tax?**

- $3000+ per week ($156,000)
- $2000-$2,999 per week ($104,000-$155,999per year)
- $1500-$1999 per week ($78,000-$103,999 per year)
- $1000-$1499 per week ($52,000-$77,999 per year)
- $600-$999 per week ($31,200-$51,999 per year)
- $200-$599 per week ($10,400-$31,199 per year)
- $1-$199 per week ($1,000-$10,399 per year)
- $0

***The following questions ask you about your health and medical history***

**15. In general would you say your health is?**

- Excellent
- Very Good
- Good
- Fair
- Poor

***The next question asks about your heart health***

**16. Has the doctor ever told you that you have had any of the following medical conditions?**

*Tick as many boxes that apply and please specify how old you were when you found out.*

- **Angina**

How old were you? _______________________

- **Heart attack**

How old were you? _______________________

***(Continue to next column)***

- **Other heart condition e.g. heart failure**

Please specify condition_____________________

_________________________________

How old were you?___________________

- **Hypertension (high blood pressure)**

How old were you? ____________________

- **High cholesterol**

How old were you? ________________________

- **High triglycerides**

How old were you? ________________________

***The next question asks about your other medical conditions?***

**17. Has the doctor ever told you that you have any of the following medical conditions?**  (*Tick ALL that apply)*

- I don’t have any other medical conditions
- Stroke
- Kidney failure or having dialysis
- Inflammatory conditions *e.g. Rheumatoid arthritis, Chron’s disease.*
- Liver failure
- Fatty liver
- Cancer (currently receiving treatment)
- Diabetes (Type 2)
- Diabetes (Type 1)
- Thyroid overactive
- Thyroid underactive
- Depression
- Other please specify______________________

**18. Have any of your immediate relatives (parents, brothers or sisters) ever been diagnosed with the following medical conditions?** (*Tick ALL that apply)*

- Angina
- Heart attack
- Stroke
- High blood pressure
- High cholesterol
- Diabetes (Type 2)
- Thyroid (either over or underactive)
- No, they do not have these medical conditions

**19. Do you currently take any prescription medications?**

- Yes
- No ***(Skip to Question 22)***

**20. Please list all the names of the prescription medications you take.**

| **Name** | **Strength** | **How many tablets per day** |
| --- | --- | --- |
| ***e.g. Lipitor*** | ***80mg*** | ***1 tablet*** |
|  |  |  |
|  |  |  |
|  |  |  |
|  |  |  |
|  |  |  |
|  |  |  |
|  |  |  |
|  |  |  |
|  |  |  |
|  |  |  |

**21. In the past month how often did you take your medication as the doctor prescribed?**

- All of the time (100%)
- Nearly all of the time (90%)
- Most of the time (75%)
- About half of the time (50%)
- Less than half of the time (<50%)

**22. Do you take any supplements e.g. vitamins, minerals, fish oil, fibre supplements etc.**

- Yes
- No ***(Skip to Question 24)***

**23. Please list ALL the supplements that you take in the table below and include brand name and dose.**

| ***Type*** | ***Brand name*** | ***Dose – How many tablets do you take per day*** |
| --- | --- | --- |
| ***E.g. Vitamin D*** | ***E.g. Ostelin*** | ***E.g. 1 per day*** |
|  |  |  |
|  |  |  |
|  |  |  |
|  |  |  |
|  |  |  |

***The following questions ask you about your smoking history***

**24. Have you ever smoked at least 100 cigarettes or the equivalent amount of tobacco in your lifetime?**

- Yes
- No ***(Skip to Question 30)***

**25. Have you ever smoked daily?** (= almost every day for at least one year)

- Yes
- No

**26. Do you now smoke?**

- Yes, daily ***(Skip to Question 28)***
- Yes, occasionally
- Not at all

**27. When did you stop smoking daily?**

- Today or yesterday
- 2 days to 6 days ago
- 1 week to less than 1 month ago
- 1 month to less than 1 year ago
- 1 to 5 years ago
- More than 5 years ago

**28. On average, what number of cigarettes do/did you smoke per day?** ___________________________

**29. How many years have you smoked/did you smoke daily?** _______________________________________

***The following questions ask you about the
kinds of physical activities that people do as part of their everyday lives.***

*The questions will ask you about the time you spent being physically active in the* ***last 7 days****. Please answer each question even if you do not consider yourself to be an active person. Please think about the activities you do at work, as part of your house and yard work, to get from place to place, and in your spare time for recreation, exercise or sport.*

Think about all the **vigorous** activities that you did in the **last 7 days**. **Vigorous** physical activities refer to activities that take hard physical effort and make you breathe much harder than normal. Think *only* about those physical activities that you did for at least 10 minutes at a time.

**30.** During the **last 7 days**, on how many days did you do **vigorous** physical activities like heavy lifting, digging, aerobics, or fast bicycling?

_____ **days per week**

- No vigorous physical activities ***(Skip to question 32)***

**31.** How much time did you usually spend doing **vigorous** physical activities on one of those days?

_____ **hours per day**

_____ **minutes per day**

- Don’t know/Not sure

**32.** Think about all the **moderate** activities that you did in the **last 7 days**. **Moderate** activities refer to activities that take moderate physical effort and make you breathe somewhat harder than normal. Think only about those physical activities that you did for at least 10 minutes at a time.

During the **last 7 days**, on how many days did you do **moderate** physical activities like carrying light loads, bicycling at a regular pace, or doubles tennis? Do not include walking.

_______ **days per week**

- No moderate physical activities ***(Skip to question 34)***

**33.** How much time did you usually spend doing **moderate** physical activities on one of those days?

_____ **hours per day**

_____ **minutes per day**

- Don’t know/Not sure

**34.** Think about the time you spent **walking** in the **last 7 days**. This includes at work and at home, walking to travel from place to place, and any other walking that you have done solely for recreation, sport, exercise, or leisure.

During the **last 7 days**, on how many days did you **walk** for at least 10 minutes at a time?

_____ **days per week**

- No walking ***(Skip to question 36)***

**35.** How much time did you usually spend **walking** on

one of those days?

_____ **hours per day**

_____ **minutes per day**

- Don’t know/Not sure

**36.** The last question is about the time you spent **sitting** on weekdays during the **last 7 days**. Include time spent at work, at home, while doing course work and during leisure time. This may include time spent sitting at a desk, visiting friends, reading, or sitting or lying down to watch television.

During the **last 7 days**, how much time did you spend

**sitting** on a **week day**?

_____ **hours per day**

_____ **minutes per day**

- Don’t know/Not sure

***You have now finished the first part of this survey. Next we would like to ask you some questions about what you like to eat and drink.***

***The following table asks you how often you have eaten certain foods over the last 12 months***

*Please estimate your average food intake as best you can, place* ***ONE*** *tick per line for* ***every food*** *in the table.*

| ***How often do you eat*** | **Never** | **Times per month** | | **Times per week** | | | | **Times per day** | | |
| --- | --- | --- | --- | --- | --- | --- | --- | --- | --- | --- |
|  |  | **1** | **2 -3** | **1** | **2** | **3 -4** | **5 -6** | **1** | **2** | **3** |
| **VEGETABLES** | | | | | | | | | | |
| **Salad vegetables** *(e.g. lettuce, cucumber etc.)* | **🞎** | **🞎** | **🞎** | **🞎** | **🞎** | **🞎** | **🞎** | **🞎** | **🞎** | **🞎** |
| **Green leafy vegetables** *(e.g. spinach, bok choy etc)* | **🞎** | **🞎** | **🞎** | **🞎** | **🞎** | **🞎** | **🞎** | **🞎** | **🞎** | **🞎** |
| **Orange & red vegetables** *(e.g. pumpkin, tomato etc.)* | **🞎** | **🞎** | **🞎** | **🞎** | **🞎** | **🞎** | **🞎** | **🞎** | **🞎** | **🞎** |
| **Potato** *excluding fried chips* | **🞎** | **🞎** | **🞎** | **🞎** | **🞎** | **🞎** | **🞎** | **🞎** | **🞎** | **🞎** |
| **All other vegetables** *(peas, cauliflower etc.)* | **🞎** | **🞎** | **🞎** | **🞎** | **🞎** | **🞎** | **🞎** | **🞎** | **🞎** | **🞎** |
| **Onion** or foods cooked with onion | **🞎** | **🞎** | **🞎** | **🞎** | **🞎** | **🞎** | **🞎** | **🞎** | **🞎** | **🞎** |
| **Garlic** or foods cooked with garlic | **🞎** | **🞎** | **🞎** | **🞎** | **🞎** | **🞎** | **🞎** | **🞎** | **🞎** | **🞎** |
| **Meals made with a tomato-based sauce** (made from fresh/paste/tinned tomatoes) | **🞎** | **🞎** | **🞎** | **🞎** | **🞎** | **🞎** | **🞎** | **🞎** | **🞎** | **🞎** |
| **Olives** | **🞎** | **🞎** | **🞎** | **🞎** | **🞎** | **🞎** | **🞎** | **🞎** | **🞎** | **🞎** |
| **Legumes** *(e.g. baked beans, lentils, chickpeas etc.)* | **🞎** | **🞎** | **🞎** | **🞎** | **🞎** | **🞎** | **🞎** | **🞎** | **🞎** | **🞎** |
| **Tofu/tempeh or vegetarian products** *(e.g. Quorn, Vegetarian sausages etc.)* | **🞎** | **🞎** | **🞎** | **🞎** | **🞎** | **🞎** | **🞎** | **🞎** | **🞎** | **🞎** |
| **FRUIT** | | | | | | | | | | |
| **Melon** *e.g. watermelon, cantaloupe etc.* | **🞎** | **🞎** | **🞎** | **🞎** | **🞎** | **🞎** | **🞎** | **🞎** | **🞎** | **🞎** |
| **Stone fruit** *e.g. apricots, nectarines etc.* | **🞎** | **🞎** | **🞎** | **🞎** | **🞎** | **🞎** | **🞎** | **🞎** | **🞎** | **🞎** |
| **Berries** *e.g. strawberries, blueberries etc.* | **🞎** | **🞎** | **🞎** | **🞎** | **🞎** | **🞎** | **🞎** | **🞎** | **🞎** | **🞎** |
| **Citrus fruit** *e.g. orange, lemon* | **🞎** | **🞎** | **🞎** | **🞎** | **🞎** | **🞎** | **🞎** | **🞎** | **🞎** | **🞎** |
| **Other fruit** *e.g. apple, pear, banana, grapes etc.* | **🞎** | **🞎** | **🞎** | **🞎** | **🞎** | **🞎** | **🞎** | **🞎** | **🞎** | **🞎** |
| **Dried fruit** *e.g. sultanas, figs etc.* | **🞎** | **🞎** | **🞎** | **🞎** | **🞎** | **🞎** | **🞎** | **🞎** | **🞎** | **🞎** |
| **MEAT & FISH** | | | | | | | | | | |
| **Fresh or canned fish** *excluding battered fish e.g. fish and chips* | **🞎** | **🞎** | **🞎** | **🞎** | **🞎** | **🞎** | **🞎** | **🞎** | **🞎** | **🞎** |
| **Red meat** i.e. beef, lamb or goat (*exclude processed meats e.g. salami, ham etc.)* | **🞎** | **🞎** | **🞎** | **🞎** | **🞎** | **🞎** | **🞎** | **🞎** | **🞎** | **🞎** |
| ***How often do you eat*** | **Never** | **Times per month** | | **Times per week** | | | | **Times per day** | | |
|  |  | **1** | **2-3** | **1** | **2** | **3 -4** | **5 -6** | **1** | **2** | **3** |
| **White meat** i.e. chicken or pork *(exclude processed meats e.g. chicken loaf, bacon etc.)* | **🞎** | **🞎** | **🞎** | **🞎** | **🞎** | **🞎** | **🞎** | **🞎** | **🞎** | **🞎** |
| **Processed meat** (e.g. sausages, bacon, salami, ham, devon etc.) | **🞎** | **🞎** | **🞎** | **🞎** | **🞎** | **🞎** | **🞎** | **🞎** | **🞎** | **🞎** |
| **Game meat** *(e.g. rabbit, venison etc.)* | **🞎** | **🞎** | **🞎** | **🞎** | **🞎** | **🞎** | **🞎** | **🞎** | **🞎** | **🞎** |
| **Game birds** *(e.g. turkey, duck, quail etc.)* | **🞎** | **🞎** | **🞎** | **🞎** | **🞎** | **🞎** | **🞎** | **🞎** | **🞎** | **🞎** |
| **Offal** *(e.g. kidney, liver, intestine etc.)* | **🞎** | **🞎** | **🞎** | **🞎** | **🞎** | **🞎** | **🞎** | **🞎** | **🞎** | **🞎** |
| **CEREAL FOODS** | | | | | | | | | | |
| **Breakfast cereal** | **🞎** | **🞎** | **🞎** | **🞎** | **🞎** | **🞎** | **🞎** | **🞎** | **🞎** | **🞎** |
| **Pasta** | **🞎** | **🞎** | **🞎** | **🞎** | **🞎** | **🞎** | **🞎** | **🞎** | **🞎** | **🞎** |
| **Rice** | **🞎** | **🞎** | **🞎** | **🞎** | **🞎** | **🞎** | **🞎** | **🞎** | **🞎** | **🞎** |
| **Noodles** | **🞎** | **🞎** | **🞎** | **🞎** | **🞎** | **🞎** | **🞎** | **🞎** | **🞎** | **🞎** |
| **Bread** | **🞎** | **🞎** | **🞎** | **🞎** | **🞎** | **🞎** | **🞎** | **🞎** | **🞎** | **🞎** |
| **Crispbreads, crackers, dry biscuits** | **🞎** | **🞎** | **🞎** | **🞎** | **🞎** | **🞎** | **🞎** | **🞎** | **🞎** | **🞎** |
| **Other grains** *(e.g. quinoa, barley, bulgar etc.)* | **🞎** | **🞎** | **🞎** | **🞎** | **🞎** | **🞎** | **🞎** | **🞎** | **🞎** | **🞎** |
| **DAIRY** | | | | | | | | | | |
| **Yoghurt** | **🞎** | **🞎** | **🞎** | **🞎** | **🞎** | **🞎** | **🞎** | **🞎** | **🞎** | **🞎** |
| **Cheese** | **🞎** | **🞎** | **🞎** | **🞎** | **🞎** | **🞎** | **🞎** | **🞎** | **🞎** | **🞎** |
| **Milk** | **🞎** | **🞎** | **🞎** | **🞎** | **🞎** | **🞎** | **🞎** | **🞎** | **🞎** | **🞎** |
| **EGGS** | | | | | | | | | | |
| **Eggs** | **🞎** | **🞎** | **🞎** | **🞎** | **🞎** | **🞎** | **🞎** | **🞎** | **🞎** | **🞎** |
| **OTHER** | | | | | | | | | | |
| **Nuts and/or seeds** | **🞎** | **🞎** | **🞎** | **🞎** | **🞎** | **🞎** | **🞎** | **🞎** | **🞎** | **🞎** |
| **Snacks** *(e.g. potato crisps, sweet/savoury biscuits, lollies, cakes and sweet pastries)* | **🞎** | **🞎** | **🞎** | **🞎** | **🞎** | **🞎** | **🞎** | **🞎** | **🞎** | **🞎** |
| **Chocolate** | **🞎** | **🞎** | **🞎** | **🞎** | **🞎** | **🞎** | **🞎** | **🞎** | **🞎** | **🞎** |
| **Meals that aren’t prepared at home** (take away and restaurant food) | **🞎** | **🞎** | **🞎** | **🞎** | **🞎** | **🞎** | **🞎** | **🞎** | **🞎** | **🞎** |
| ***How often do you eat*** | **Never** | **Times per month** | | **Times per week** | | | | **Times per day** | | |
|  |  | **1** | **2 -3** | **1** | **2** | **3 -4** | **5 -6** | **1** | **2** | **3** |
| **HERBS & SPICES** | | | | | | | | | | |
| **Oregano (fresh or dried)** | **🞎** | **🞎** | **🞎** | **🞎** | **🞎** | **🞎** | **🞎** | **🞎** | **🞎** | **🞎** |
| **Curry powder** | **🞎** | **🞎** | **🞎** | **🞎** | **🞎** | **🞎** | **🞎** | **🞎** | **🞎** | **🞎** |
| **Turmeric** | **🞎** | **🞎** | **🞎** | **🞎** | **🞎** | **🞎** | **🞎** | **🞎** | **🞎** | **🞎** |
| **Cinnamon** | **🞎** | **🞎** | **🞎** | **🞎** | **🞎** | **🞎** | **🞎** | **🞎** | **🞎** | **🞎** |
| **Ginger** | **🞎** | **🞎** | **🞎** | **🞎** | **🞎** | **🞎** | **🞎** | **🞎** | **🞎** | **🞎** |
| **Chilli (fresh or dried)** | **🞎** | **🞎** | **🞎** | **🞎** | **🞎** | **🞎** | **🞎** | **🞎** | **🞎** | **🞎** |
| **CONDIMENTS ADDED TO MEALS** | | | | | | | | | | |
| **Lemon juice** | **🞎** | **🞎** | **🞎** | **🞎** | **🞎** | **🞎** | **🞎** | **🞎** | **🞎** | **🞎** |
| **Vinegar (any type)** | **🞎** | **🞎** | **🞎** | **🞎** | **🞎** | **🞎** | **🞎** | **🞎** | **🞎** | **🞎** |
| **Tomato sauce** | **🞎** | **🞎** | **🞎** | **🞎** | **🞎** | **🞎** | **🞎** | **🞎** | **🞎** | **🞎** |
| **Soy sauce** | **🞎** | **🞎** | **🞎** | **🞎** | **🞎** | **🞎** | **🞎** | **🞎** | **🞎** | **🞎** |
| **Salt** | **🞎** | **🞎** | **🞎** | **🞎** | **🞎** | **🞎** | **🞎** | **🞎** | **🞎** | **🞎** |
| **Pepper** | **🞎** | **🞎** | **🞎** | **🞎** | **🞎** | **🞎** | **🞎** | **🞎** | **🞎** | **🞎** |

***The following questions ask you about how much you eat of certain foods over the last 12 months.***

*Please estimate the amount you eat on average as best as you can using the following pictures as a guide and attempt to answer every question by placing a tick in the box.*

| **1. On average when you eat *salad vegetables*, do you eat?**  🞎 I don’t eat salad vegetables  **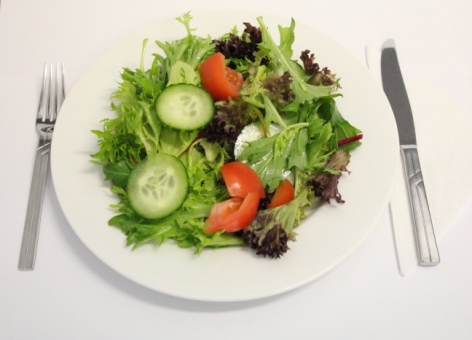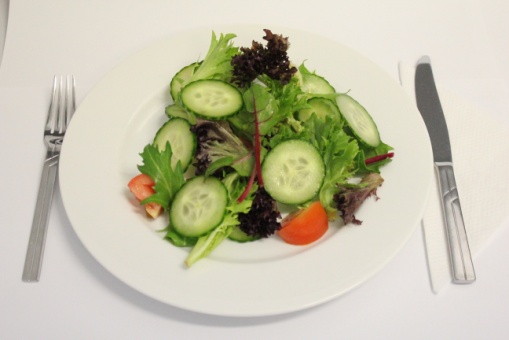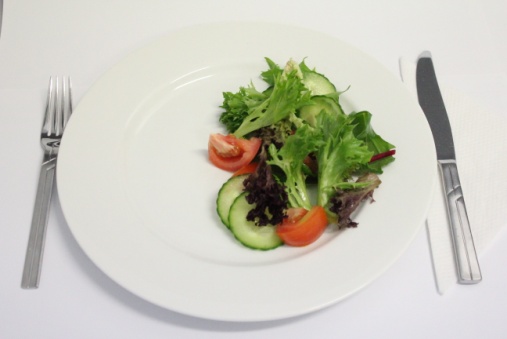** |
| --- |
| 🞎 🞎 🞎 🞎 🞎 🞎 🞎  Less than A A Between A & B B Between B & C C More than C  C  B  A |
| **2. On average when you eat *green leafy vegetables*, do you eat?**  🞎 I don’t eat green leafy vegetables  **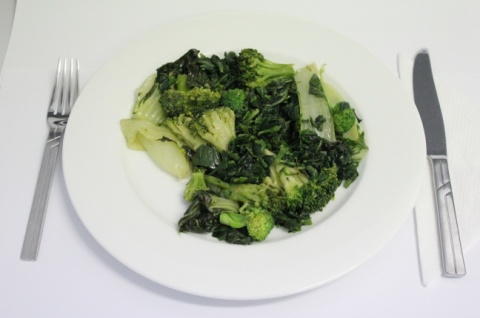**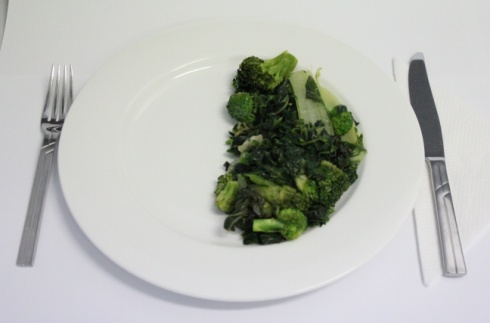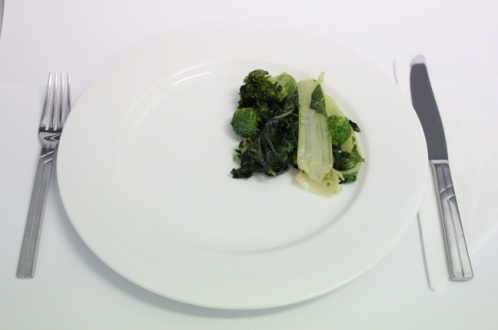  C  B  A |
| 🞎 🞎 🞎 🞎 🞎 🞎 🞎  Less than A A Between A & B B Between B & C C More than C |
| **3. On average when you eat *potato,* do you eat?**  🞎 I don’t eat potato  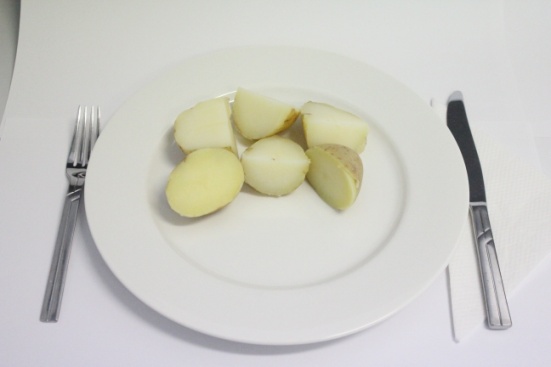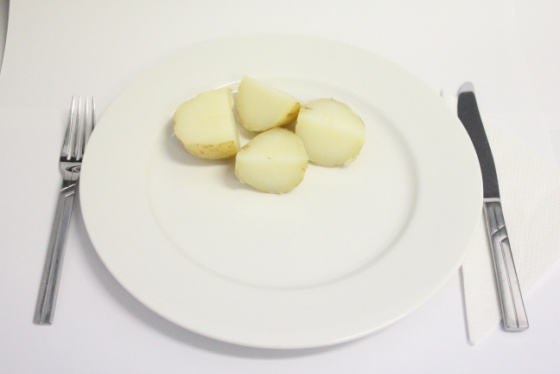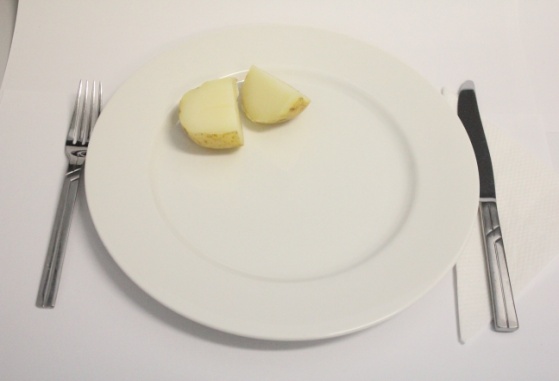  A  C  B |
| 🞎 🞎 🞎 🞎 🞎 🞎 🞎  Less than A A Between A & B B Between B & C C More than C |
| **4. On average when you eat *vegetables*, do you eat?**  🞎 I don’t eat vegetables  **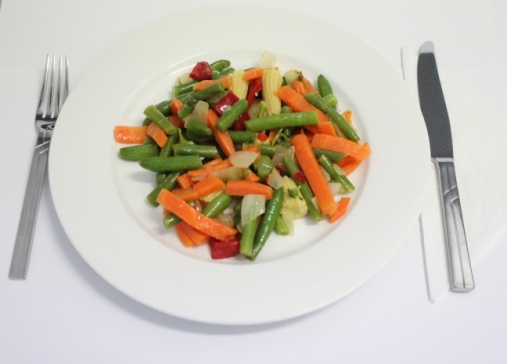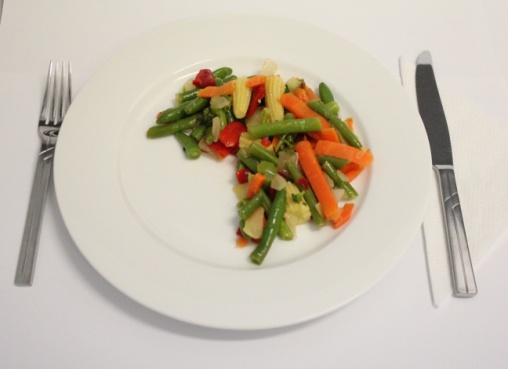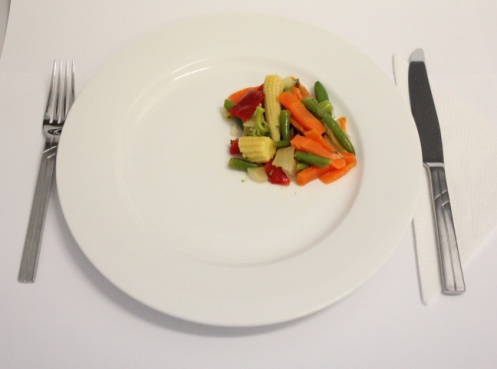**  C  A |
| 🞎 🞎 🞎 🞎 🞎 🞎 🞎  B  Less than A A Between A & B B Between B & C C More than C |
| **5. On average when you eat *legumes*, do you eat?**  🞎 I don’t eat legumes  **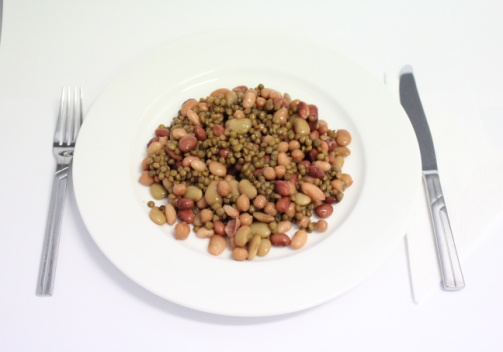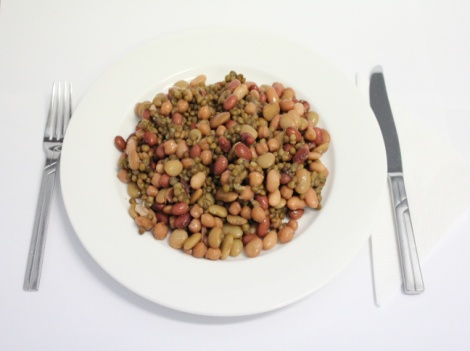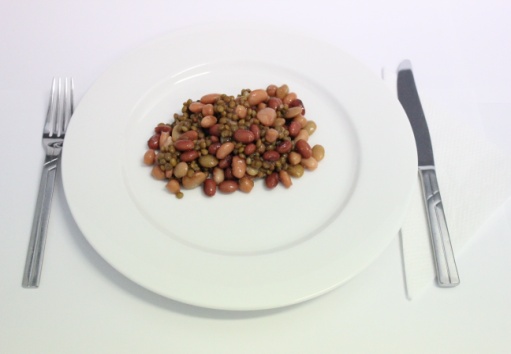** |
| 🞎 🞎 🞎 🞎 🞎 🞎 🞎  C  B  A  Less than A A Between A & B B Between B & C C More than C |
| **6. On average when you eat *fish*, do you eat?**  🞎 I don’t eat fish  **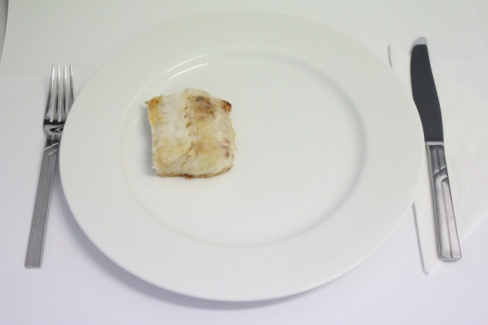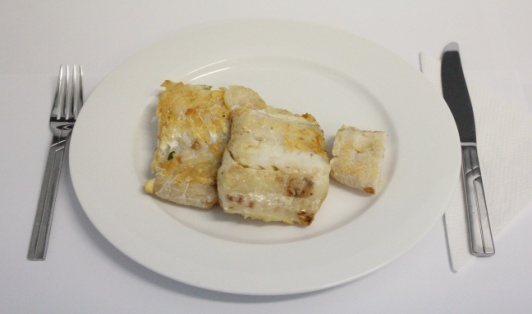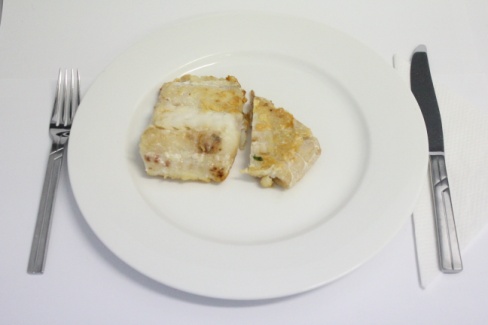**  C  B  A |
| 🞎 🞎 🞎 🞎 🞎 🞎 🞎  Less than A A Between A & B B Between B & C C More than C |
| **7. On average when you eat *red meat,* do you eat? (Include meat from mixed dishes e.g. casseroles)**  🞎 I don’t eat red meat  B  C  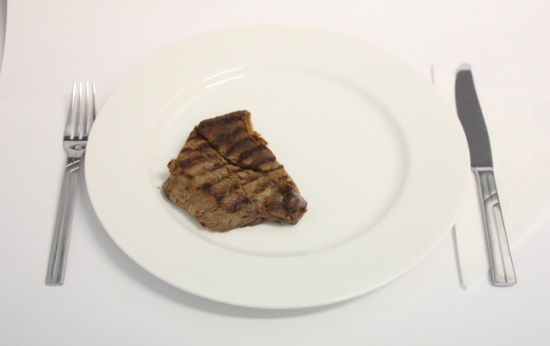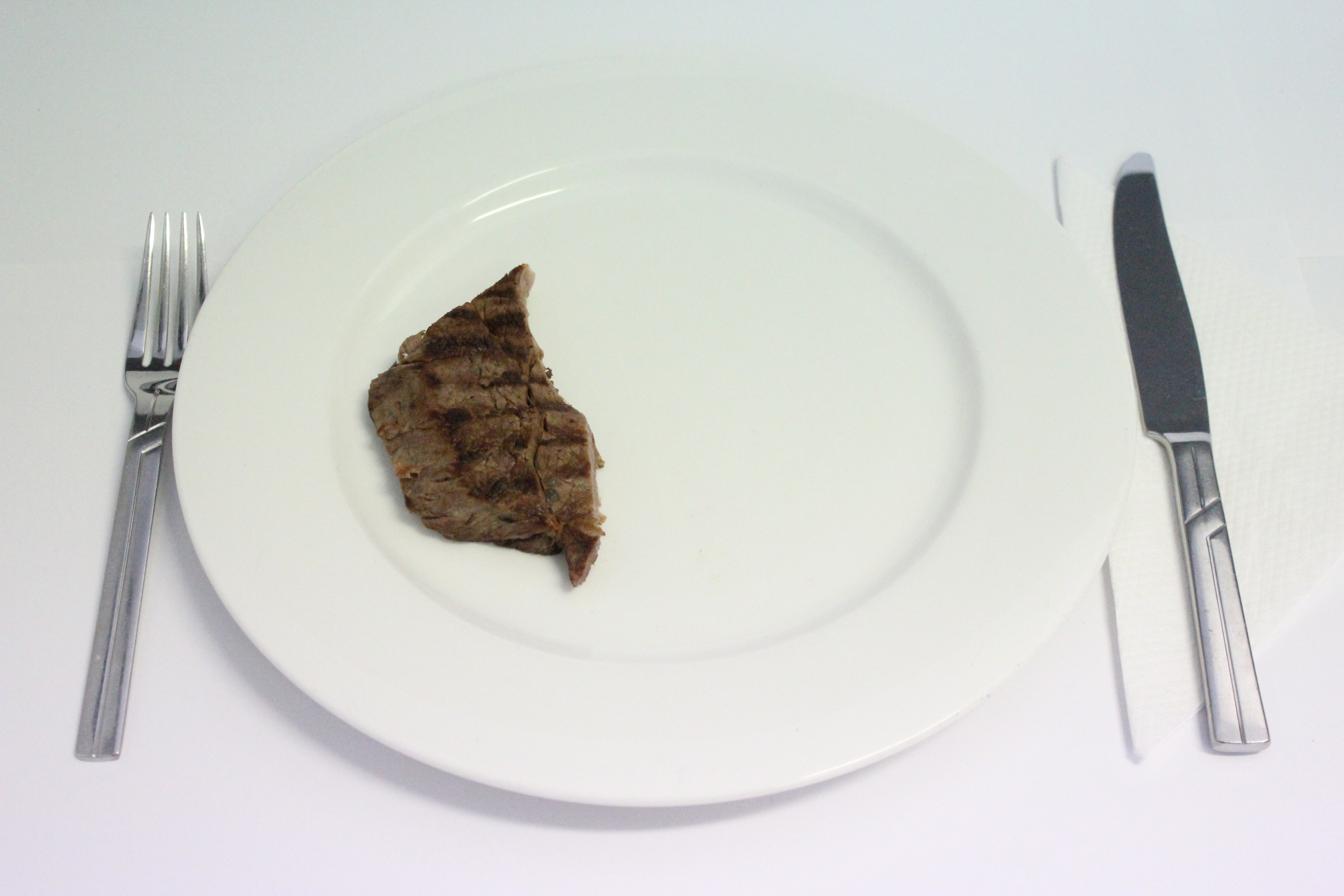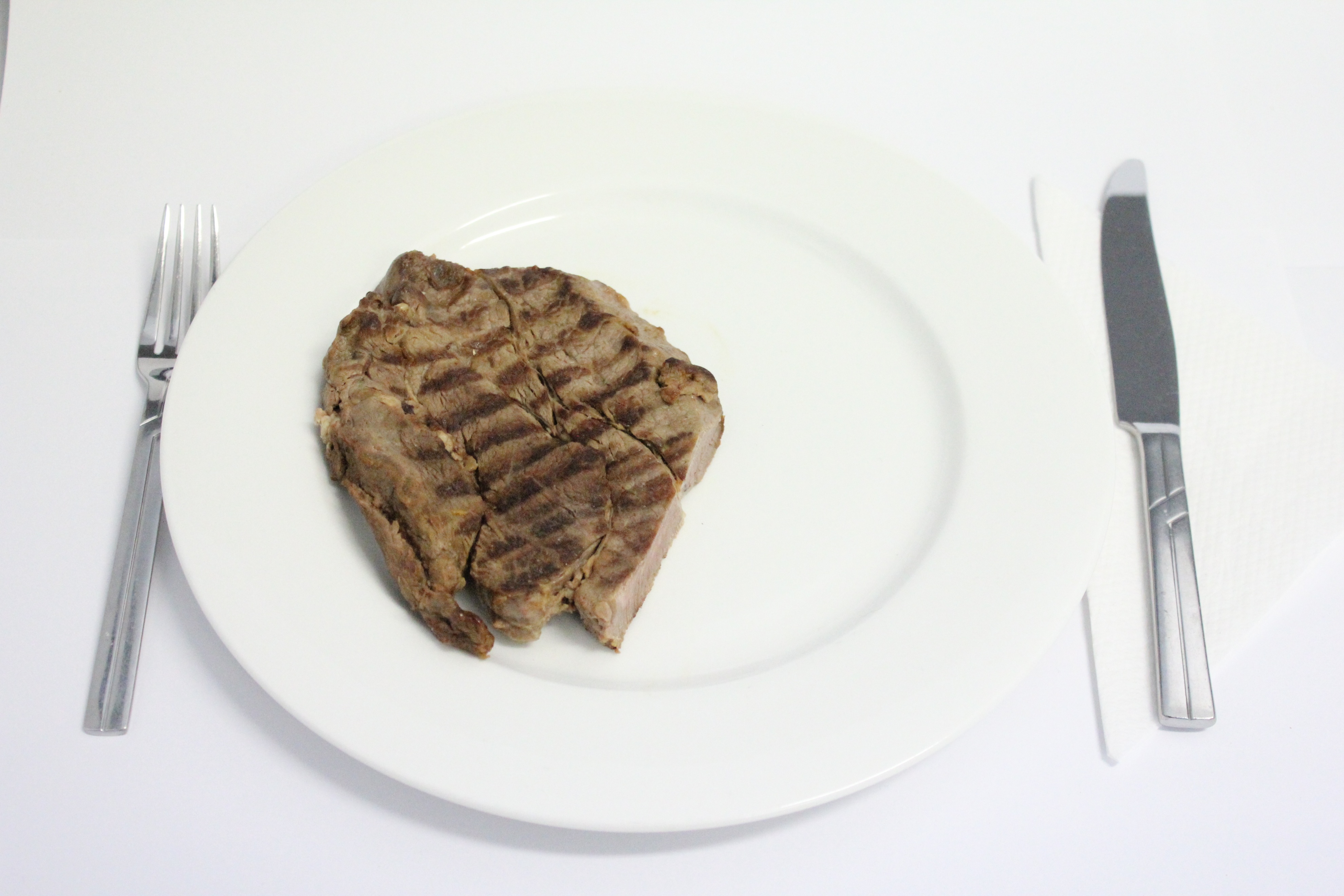  A |
| 🞎 🞎 🞎 🞎 🞎 🞎 🞎  Less than A A Between A & B B Between B & C C More than C |
| **8. On average when you eat *white meat*, do you eat? (Include meat from mixed dishes e.g. casseroles)**  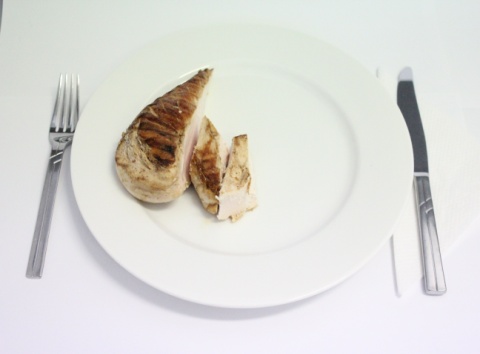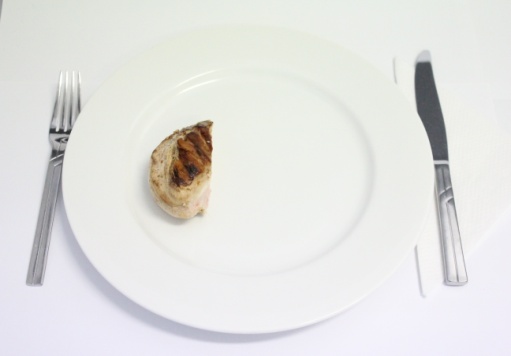 🞎 I don’t eat white meat  C  **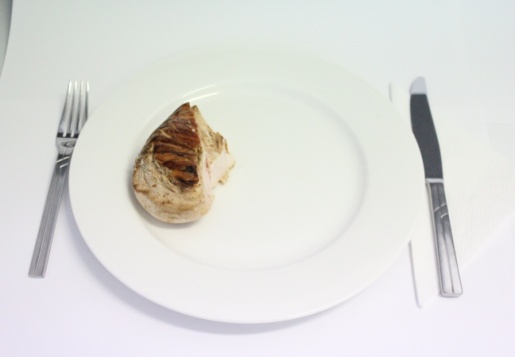** |
| 🞎 🞎 🞎 🞎 🞎 🞎 🞎  B  A  Less than A A Between A & B B Between B & C C More than C |
| **9. On average when you eat *breakfast cereal*, do you eat?**  🞎 I don’t eat breakfast cereal |
| 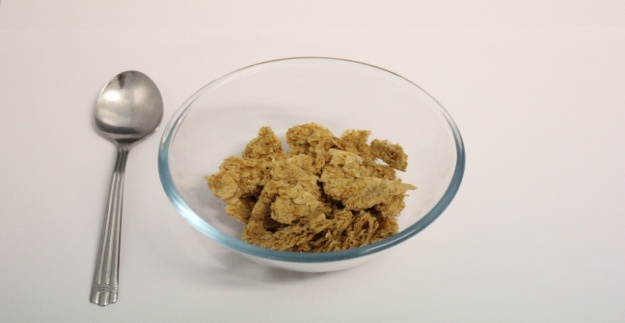  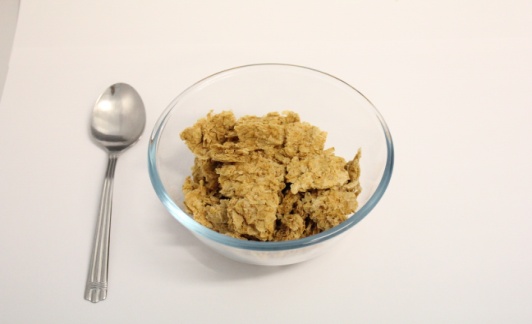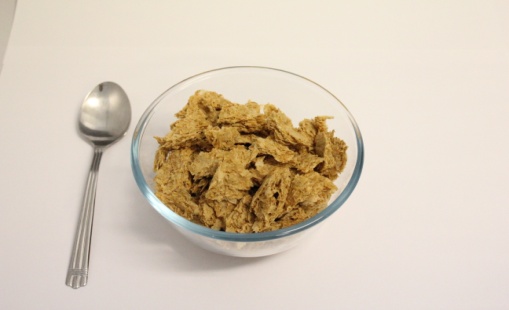 🞎 🞎 🞎 🞎 🞎 🞎 🞎  C  B  A  Less than A A Between A & B B Between B & C C More than C |
| **10. On average when you eat *rice*, do you eat?**  🞎 I don’t eat rice |
| 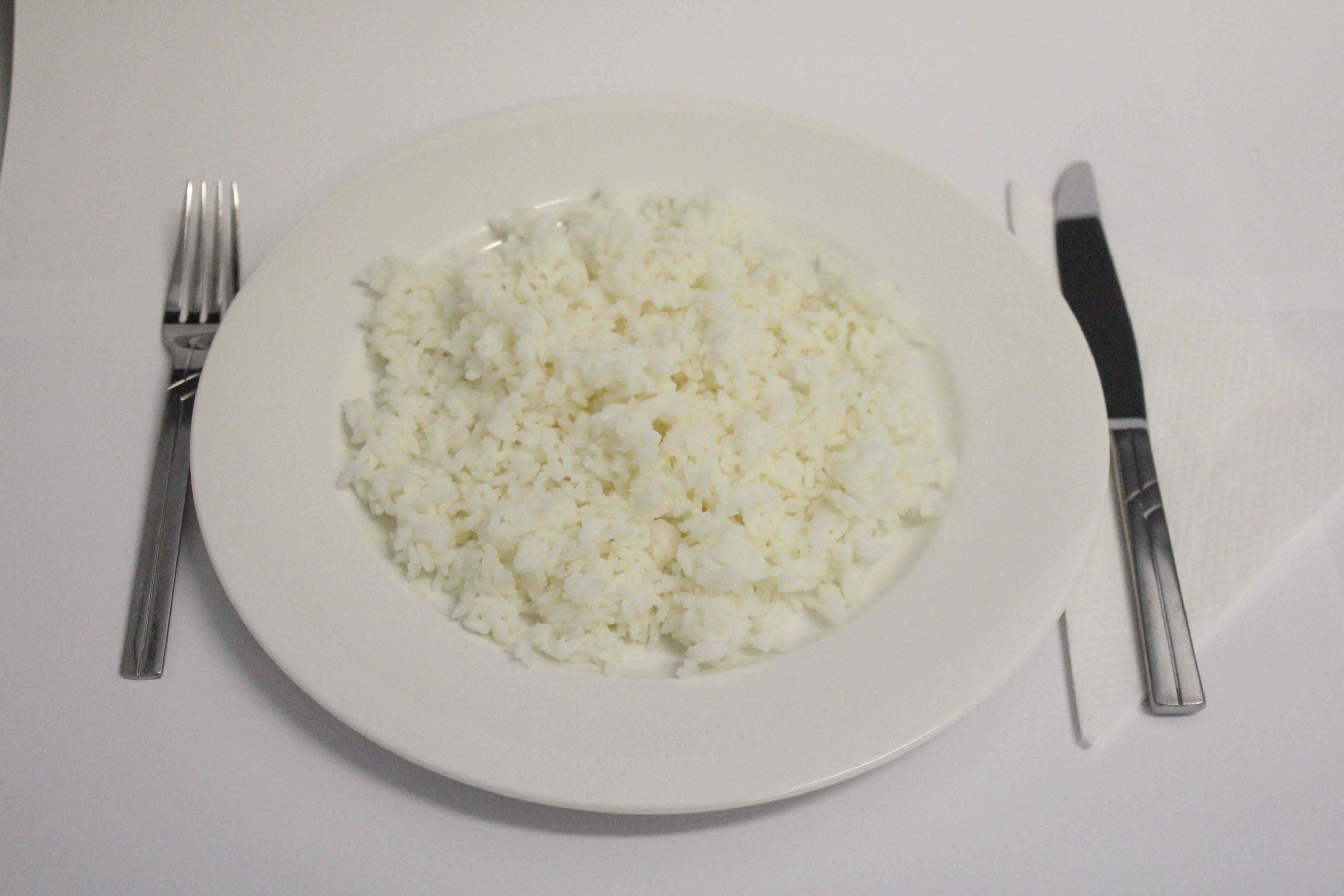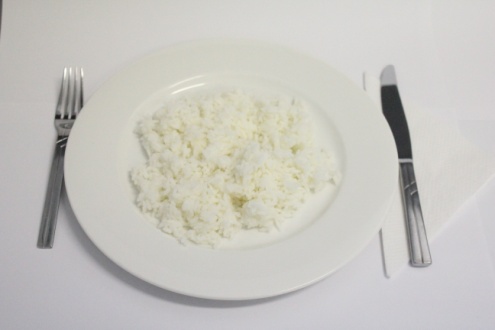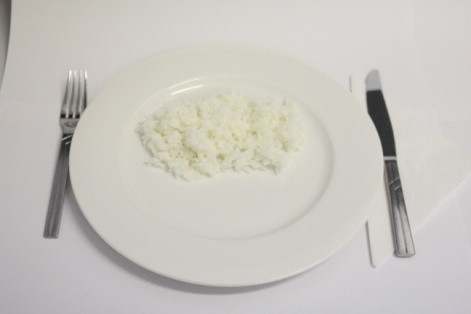 🞎 🞎 🞎 🞎 🞎 🞎 🞎  A  C  B  Less than A A Between A & B B Between B & C C More than C |
| **11. On average when you eat *pasta,* do you eat?**  🞎 I don’t eat pasta  **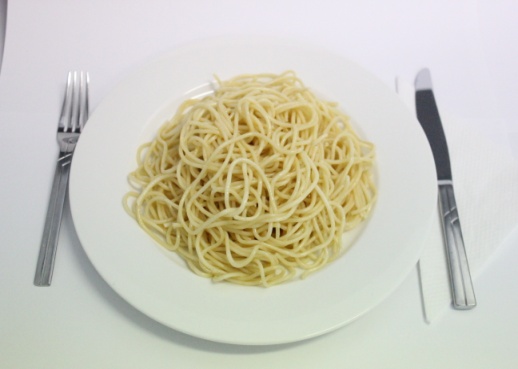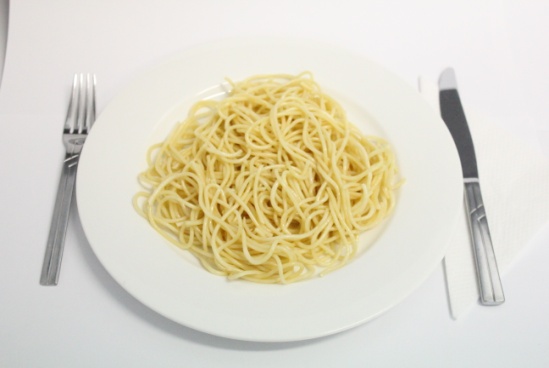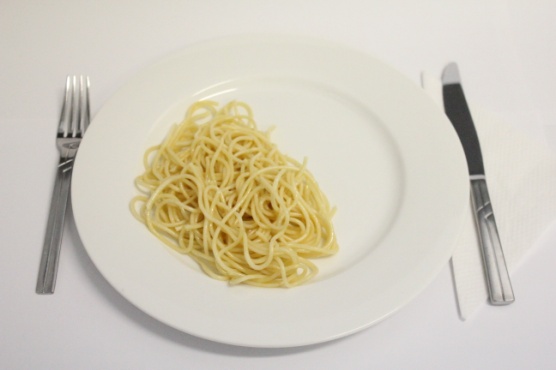** |
| 🞎 🞎 🞎 🞎 🞎 🞎 🞎  C  B  A  Less than A A Between A & B B Between B & C C More than C |
| **12. On average when you eat *noodles*, do you eat?**   🞎 I don’t eat noodles  **** 🞎 🞎 🞎 🞎 🞎 🞎 🞎  C  B  A  Less than A A Between A & B B Between B & C C More than C  **13. On average when you eat *yoghurt,* do you eat?**  🞎 I don’t eat yoghurt |
| **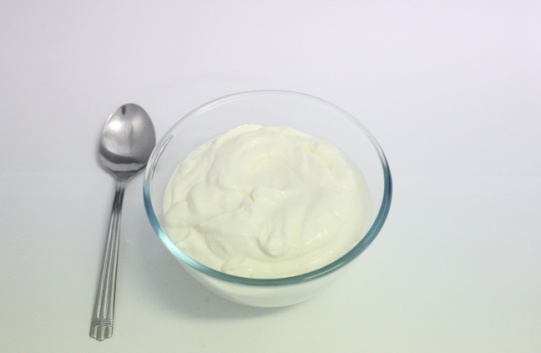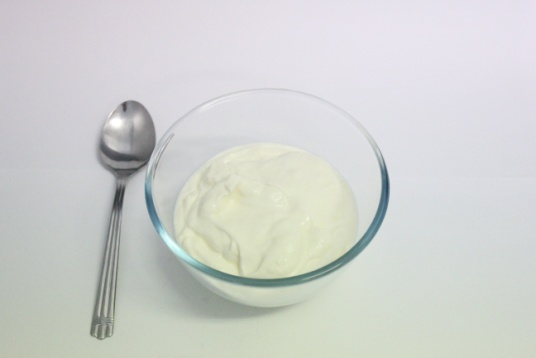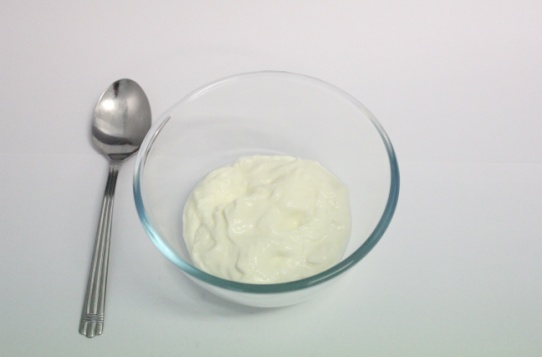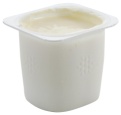**🞎 🞎 🞎 🞎 🞎 🞎 🞎  Less than A A Between A & B B Between B & C C More than C  **=**  C  B  A |
| **14. On average when you eat *cheese,* do you eat?**  🞎 I don’t eat cheese |
| 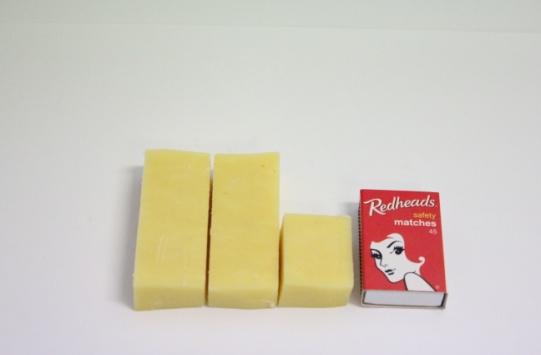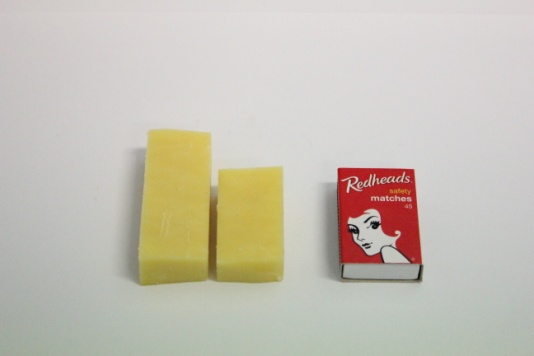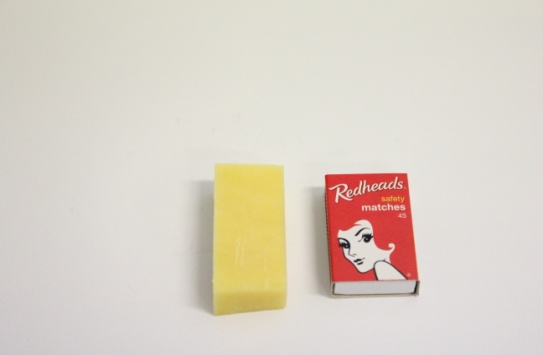🞎 🞎 🞎 🞎 🞎 🞎 🞎  C  B  A  Less than A A Between A & B B Between B & C C More than C |
| **15. On average, when you eat *nuts*, do you eat?**  🞎 I don’t eat nuts |
| 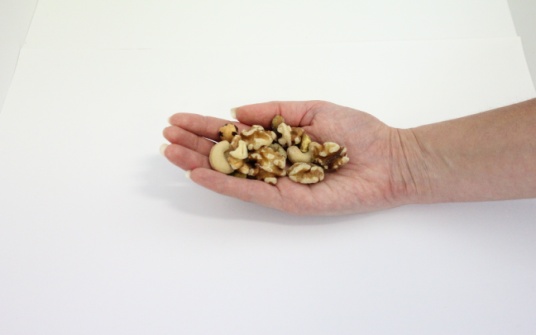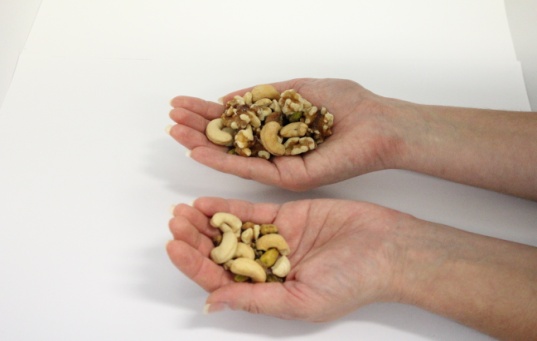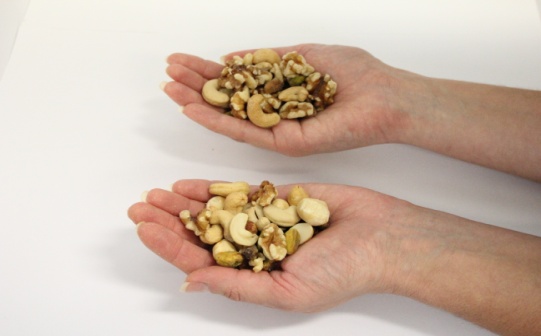🞎 🞎 🞎 🞎 🞎 🞎 🞎  B  C  A  A  Less than A A Between A & B B Between B & C C More than C |

***The following questions ask you about the types of food that you prefer to eat and your dietary habits over the last 12 months.***

*Please estimate your average food intake as best as you can and please attempt to answer every question*

**1. What is the MAIN type of fat or oil that you use for COOKING?** (*Please tick ONE option)*

- I don’t use anything
- Extra virgin olive oil
- Other olive oil *e.g. light*
- Canola, peanut, sesame, rice bran oil
- Vegetable, sunflower, safflower, grapeseed oil
- Ghee, butter, palm oil, lard, dripping
- Coconut oil

Margarine

- Monounsaturated *e.g. Olive Grove, Meadow Lea*
- Polyunsaturated *e.g. Flora, Nuttelex*
- Cholesterol lowering *e.g. Flora Pro Activ, Meadow Lea Logicol*
- Margarine and butter blend

**2. What is the MAIN type of fat or oil that you add to COOKED VEGETABLES**? (*Please tick ONE option)*

- I don’t use anything
- Extra virgin olive oil
- Other olive oil *e.g. light*
- Canola, peanut, sesame, rice bran oil
- Vegetable, sunflower, safflower, grapeseed oil
- Ghee, butter, palm oil, lard, dripping
- Coconut oil

Margarine

- Monounsaturated *e.g. Olive Grove, Meadow Lea*
- Polyunsaturated *e.g. Flora, Nuttelex*
- Cholesterol lowering *e.g. Flora Pro Activ, Meadow Lea Logicol*
- Margarine and butter blend

**3. What type of dressings do you usually add to SALAD vegetables?** *(Tick ALL that apply)*

- No dressing
- Extra virgin olive oil

***(Continue to next column)***

- Other olive oil *e.g. light*
- Canola, peanut, sesame, rice bran oil
- Vegetable, sunflower, safflower, grapeseed oil
- Full fat commercial salad dressings
- Reduced fat commercial salad dressings
- Vinegar
- Mayonnaise
- Lemon juice

**4. What is the MAIN type of spread that you usually put on BREAD?** (*Please tick ONE option)*

- I don’t use anything

Margarine

- Monounsaturated *e.g. Olive Grove,*

*Meadow Lea*

- Polyunsaturated *e.g. Flora, Nuttelex*
- Cholesterol lowering *e.g. Flora Pro Activ, Meadow Lea Logicol*
- Margarine and butter blend

🞎 Butter

🞎 Nut spreads *e.g. peanut butter, tahini*

**5. How much fat and/or oil (in total) do you consume on average per day?** *(Think about all the types of fat and/or oil you use daily e.g. in cooking/spreads on bread etc.)*

- I don’t consume fat and/or oil
- 1 teaspoon (5mL)
- 1 tablespoon (20mL)
- 2 tablespoons (40mL)
- 3 tablespoons (60mL)
- Other, please specify how much___________

**6. How often is the meat that you eat trimmed of fat before or after cooking?** (*Please tick ONE option)*

- Never/ rarely
- Sometimes
- Usually
- Always

**7. How many teaspoons of sugar do you add to your food and drink each day?** *(Include sugar in tea, coffee, cereal etc.)* (*Please tick ONE option)*

- I don’t add sugar 🞎 7-8 teaspoons
- 1-2 teaspoons 🞎 9-10 teaspoons
- 3-4 teaspoons 🞎 11-12 teaspoons
- 5-6 teaspoons 🞎 13+ teaspoons

**8. What is the MAIN type of milk that you usually drink?** *(Please tick ONE* *option)*

- I don’t drink milk ***(Skip to Question 11)***
- Cow’s milk
- Goat’s milk
- Evaporated milk
- Soy milk
- Rice milk
- Other, please specify_____________________

**9. What is the fat content of the MAIN type of milk you usually drink?** *(Please tick ONE* *option)*

- Regular full cream
- Reduced fat milk
- Skim milk
- I don’t know

**10. On average how much milk do you usually drink per day?** *(Include milk added to tea, coffee, cereal etc)*

- Less than 250ml (less than 1 cup)
- 250ml (1 cup)
- Between 250 and 500ml (1-2 cups)
- Between 500 and 750ml (2-3 cups)
- 750ml (3 cups) or more

**11. What is the MAIN type of yoghurt that you usually eat?** *(Please tick ONE* *option)*

- I don’t eat yoghurt ***(Skip to Question 13)***
- Natural
- Greek
- Vanilla
- Fruit

**12. What is the fat content of the MAIN type of yoghurt you usually eat?** (*Please tick ONE option)*

- Regular full fat
- Low fat
- I don’t know

**13. What is the MAIN type of CHEESE that you usually eat?** (*Please tick ONE option)*

- I don’t eat cheese **(*Skip to Question 15)***
- White cheese *e.g. fetta*
- Hard cheeses *e.g. parmesan*
- Firm cheeses *e.g. cheddar and edam*
- Processed cheese *e.g.cheese slices*
- Soft cheeses *e.g. camembert and brie*
- Ricotta or cottage cheese
- Cream cheese

**14. What is the fat content of the MAIN type of cheese you usually consume?** *(Please tick ONE* *option)*

- Regular full fat
- Low fat
- I don’t know

**15. On average how many eggs do you eat per week?** (*Please tick ONE option)*

- I don’t eat eggs
- Less than one egg
- 1 egg
- 1 - 2 eggs
- 3 - 5 eggs
- 6 or more eggs

**16. On average how many pieces of fresh fruit do you usually eat per day?** *(1 piece = 1 cup of diced fruit or 2 small pieces of fruit)* (*Please tick ONE option)*

- I don’t eat fruit
- Less than 1 piece
- 1 piece
- 2 pieces
- 3 pieces
- More than 3 pieces, please specify__________

**17. What is the MAIN type of bread you usually eat?** (*Please tick ONE option)*

- I don’t eat bread ***(Skip to Question 19)***
- Hi fibre white bread
- White bread
- Wholemeal bread
- Multi-grain bread
- Rye bread
- Flat bread (e.g. Roti, Pita, Wraps, Turkish etc.)
- Other, please specify_____________________

**18. On average how many slices of bread do you usually eat per day?** *(Include all types, fresh or toasted and count one bread roll/flat bread as 2 slices)*

(*Please tick ONE option)*

- Less than 1 slice per day
- 1 slice per day
- 2 slices per day
- 3 slices per day
- 4 slices per day
- 5-7 slices per day
- 8 or more slices per day

**19. What type of cereal do you usually consume?** (*Please tick ONE option)*

- I don’t eat cereal
- Porridge
- Wholegrain (e.g Muesli, Weetbix, Hi-Fibre flakes)
- Other (e.g. Cornflakes, Coco Pops, Nutri-grain)

**20. What is the MAIN type of fish you usually eat?** *(You can tick up to TWO options)*

- I don’t eat fish
- Canned fish
- White flesh fish *e.g. flake, whiting etc.*
- Oily fish *e.g. salmon, tuna, sardines etc.*
- Shellfish *e.g. prawns, lobster, crab etc.*
- Molluscs *e.g. calamari, oyster, octopus etc.*
- Other, please specify_________________

**21. What are the MAIN types of nuts and/or seeds that you usually eat?** (*Tick ALL that apply)*

- I don’t eat nuts and seeds ***(Skip to Question 23)***
- Almonds
- Walnuts
- Peanuts
- Pistachio
- Sunflower seeds
- Pepitas (pumpkin seeds)
- Other, please specify _________________

**22. Are the main type of nuts that you eat**

(*Please tick ONE option)*

- Raw / Natural
- Roasted
- Salted
- Sugar coated
- Chocolate coated

**23. When you eat foods that aren’t prepared at home (takeaway and restaurant food), which of these foods do you USUALLY choose?** (*Tick ALL that apply)*

- I never eat takeaway foods
- Meat pie, pastie, quiche & savoury pastries
- Hamburger with a bun
- Chicken and chips
- Battered/fried fish (include takeaway)
- Pasta
- Stir-fry
- Curry
- Kebab
- Pizza
- Dim sims or spring rolls
- Hot dog
- Hot chips, wedges, french fries
- Sushi
- Other, please specify_________________

**24. When you eat chocolate, which type of chocolate do you USUALLY choose?** (*Please tick ONE option)*

- I don’t eat chocolate
- Milk chocolate
- Dark chocolate
- White chocolate

**25. When you eat snack food, which of these foods do you USUALLY choose?** *(Tick ALL that apply)*

- I never eat snack foods
- Corn chips, potato chips, twisties etc.
- Popcorn
- Sweet biscuits
- Cakes or sweet pastries
- Puddings
- Confectionary containing chocolate
- Lollies
- Ice cream
- Muesli bar
- Muffin
- Other, please specify_________________

**26. What type of coffee do you MOST OFTEN drink?** *(Please tick ONE option)*

- I don’t drink coffee
- Instant
- Greek/Turkish
- Regular cappuccino / latte
- Filter
- Espresso
- Plunger
- Other, please specify ___________________

**27. What type of tea do you MOST OFTEN drink?**

*(Please* *tick ONE option)*

- I don’t drink tea
- Black Tea leaves *e.g. Lipton, Bushells* ***(tick this option even if you add milk to your tea)***
- Green Tea
- Other, please specify______________________

**28. What is the MAIN method you use for cooking vegetables?** *(You can tick up to TWO options)*

- I don’t eat cooked vegetables
- Barbeque / Grill
- Deep frying
- Fry pan / Stir fry
- Casserole / Curry
- Steaming / Boiling / Microwaving
- Roasting / Baking

**29. What is the MAIN method you use for cooking fish?** *(You can tick up to TWO options)*

- I don’t eat fish
- Barbeque / Grill
- Deep frying
- Fry pan / Stir fry
- Casserole / Curry
- Steaming / Poaching
- Roasting / Baking

**30. What is the MAIN method you use for cooking meat?** *(You can tick up to TWO options)*

- I don’t eat meat
- Barbeque / Grill
- Deep frying
- Fry pan / Stir fry
- Casserole / Curry
- Steaming / Poaching
- Roasting / Baking

**31. How often do you add salt to your food after it is cooked?** (*Please tick ONE option)*

- Never/rarely
- Sometimes
- Usually

**32. How often is salt added to your food during cooking?** (*Please tick ONE option)*

- Never/rarely
- Sometimes
- Usually

**33. Who does the food shopping in your household?** *(Tick ALL that apply)*

- I do
- Family member
- Other, please specify who_________________

**34. Who does the cooking in your household?**

*(Tick ALL that apply)*

- I do
- Family member
- Other, please specify who_________________

**35. In the last 12 months have you been on a special diet for any of these reasons?**

*(Tick ALL that apply)*

- No, I haven’t been on a special diet
- High blood pressure
- Stomach problems *e.g. ulcer, reflux*
- Bowel problems *e.g. IBS*
- Allergies *e.g. skin rash*
- Concern over a family history or illness
- High blood cholesterol
- Overweight or obesity
- Diabetes
- Concern over eating a healthy diet
- Other ________________________________

***The following questions ask you about the type of drinks regularly consumed over last 12 months. 36.*** *Please estimate how many glasses or cups you drink of the following drinks* ***-*** ***1 glass or cup = 250mL***

| ***How often do you drink*** | **Never** | **Glasses per month** | | **Glasses per week** | | | | **Glasses per day** | | | | |
| --- | --- | --- | --- | --- | --- | --- | --- | --- | --- | --- | --- | --- |
|  |  | **1** | **2 -3** | **1** | **2** | **3 -4** | **5 -6** | **1** | **2** | **3-4** | **5-6** | **7-8** |
| ***1 glass or cup = 250mL*** | | | | | | | | | | | | |
| **Water** | **🞎** | **🞎** | **🞎** | **🞎** | **🞎** | **🞎** | **🞎** | **🞎** | **🞎** | **🞎** | **🞎** | **🞎** |
| **Coffee** | **🞎** | **🞎** | **🞎** | **🞎** | **🞎** | **🞎** | **🞎** | **🞎** | **🞎** | **🞎** | **🞎** | **🞎** |
| **Tea** | **🞎** | **🞎** | **🞎** | **🞎** | **🞎** | **🞎** | **🞎** | **🞎** | **🞎** | **🞎** | **🞎** | **🞎** |
| **Pure fruit juice** | **🞎** | **🞎** | **🞎** | **🞎** | **🞎** | **🞎** | **🞎** | **🞎** | **🞎** | **🞎** | **🞎** | **🞎** |
| **Regular fizzy soft drink** | **🞎** | **🞎** | **🞎** | **🞎** | **🞎** | **🞎** | **🞎** | **🞎** | **🞎** | **🞎** | **🞎** | **🞎** |
| **Regular cordial** | **🞎** | **🞎** | **🞎** | **🞎** | **🞎** | **🞎** | **🞎** | **🞎** | **🞎** | **🞎** | **🞎** | **🞎** |
| **Diet drinks** *e.g. cordial, fizzy soft drink* | **🞎** | **🞎** | **🞎** | **🞎** | **🞎** | **🞎** | **🞎** | **🞎** | **🞎** | **🞎** | **🞎** | **🞎** |
| **Sports drink** *e.g. Gatorade* | **🞎** | **🞎** | **🞎** | **🞎** | **🞎** | **🞎** | **🞎** | **🞎** | **🞎** | **🞎** | **🞎** | **🞎** |
| **Flavoured milk** *e.g. Milo, Big M* | **🞎** | **🞎** | **🞎** | **🞎** | **🞎** | **🞎** | **🞎** | **🞎** | **🞎** | **🞎** | **🞎** | **🞎** |
| **Hot chocolate** *e.g. made with cocoa powder* | **🞎** | **🞎** | **🞎** | **🞎** | **🞎** | **🞎** | **🞎** | **🞎** | **🞎** | **🞎** | **🞎** | **🞎** |

***Finally, we would like to ask you some questions about alcohol.***

**37. How many alcoholic drinks do you drink on average each week?**

*Please put a number in the box* *below*

*(****HINT****: one drink = 1 glass of wine, 1 pot/stubbie/can of beer, 1 nip of spirits or 1 pre-mixed drink)*

🞎 drinks

***If you put “0” – You have now finished, thank you!***

**38. If you have more than one drink of alcohol each week, is it usually with meals?** *(Please tick ONE option)*

- Yes
- No
- It varies

**39. On how many days each week do you usually drink?** *(Please tick ONE option)*

- 1 day 🞎 5 days
- 2 days 🞎 6 days
- 3 days 🞎 Everyday
- 4 days

**40. What type of alcohol is your MAIN drink, the one you drink most often?** *(Please tick ONE option)*

- Red wine
- White wine
- Regular strength beer
- Low alcohol beer
- Pre-mixed spirits
- Bottled spirits and liqueurs
- Cider
- Other, please specify____________________

***You have now finished the questionnaire***
