# Supplementary material for: The assessment of dietary carotenoid intake of the Cardio-Med FFQ using food records and biomarkers in an Australian cardiology cohort: a pilot validation
Source: J Nutr Sci. 2024 Apr 11;13:e20. doi: 10.1017/jns.2024.6 (PMC11016364; doi:10.1017/jns.2024.6)
Supplement: Kucianski et al. supplementary material 2 — Kucianski et al. supplementary material [file S2048679024000065sup002.docx]

**Supplementary Materials 2**

**Table S1: Spearman’s Correlation coefficients (**$\rho$**) for confounding variables and crude carotenoids measured by FFQ (n=39)**

| **Carotenoid** | **Age** | | **Gender** | | **BMI** | | **Supplement use**^†^ | | **Smoking status**^‡^ | |
| --- | --- | --- | --- | --- | --- | --- | --- | --- | --- | --- |
|  | $\rho$ | p-value | $\rho$ | p-value | $\rho$ | p-value | $\rho$ | p-value | $\rho$ | p-value |
| **β-Carotene** | -0.12 | 0.48 | 0.06 | 0.71 | -0.06 | 0.70 | -0.01 | 0.96 | -0.27 | 0.10 |
| **α-Carotene** | 0.25 | 0.13 | -0.10 | 0.54 | -0.30 | 0.07 | 0.07 | 0.68 | -0.20 | 0.22 |
| **β-cryptoxanthin** | 0.08 | 0.62 | -0.23 | 0.16 | -0.06 | 0.70 | -0.04 | 0.80 | -0.34 | 0.03* |
| **Lycopene** | 0.09 | 0.57 | -0.14 | 0.41 | -0.19 | 0.26 | -0.01 | 0.93 | -0.12 | 0.47 |
| **Lutein/Zeaxanthin** | -0.08 | 0.64 | 0.02 | 0.90 | -0.02 | 0.91 | 0.06 | 0.74 | -0.25 | 0.13 |
| **Total Carotenoids** | -0.05 | 0.75 | 0.01 | 0.97 | -0.07 | 0.689 | 0.01 | 0.96 | -0.27 | 0.09 |

$\rho$, Spearman’s correlation coefficient; FFQ, food frequency questionnaire; BMI, body mass index.

^*^ Statistically significant correlation coefficient, p<0.05.

† Supplement use (yes/no).

‡ Smoking status (yes/no).

**Table S2: Spearman’s Correlation coefficients (**$\rho$**) for confounding variables and crude carotenoids measured by 7DFR (n=39)**

| **Carotenoid** | **Age** | | **Gender** | | **BMI** | | **Supplement use**^†^ | | **Smoking status**^‡^ | |
| --- | --- | --- | --- | --- | --- | --- | --- | --- | --- | --- |
|  | $\rho$ | p-value | $\rho$ | p-value | $\rho$ | p-value | $\rho$ | p-value | $\rho$ | p-value |
| **β-Carotene** | -0.03 | 0.87 | -0.27 | 0.10 | 0.17 | 0.31 | 0.17 | 0.32 | -0.20 | 0.23 |
| **α-Carotene** | 0.17 | 0.29 | 0.29 | 0.07 | 0.02 | 0.92 | 0.22 | 0.19 | 0.24 | 0.14 |
| **β-cryptoxanthin** | -0.01 | 0.96 | -0.06 | 0.71 | 0.05 | 0.76 | -0.01 | 0.93 | -0.19 | 0.26 |
| **Lycopene** | 0.02 | 0.91 | -0.04 | 0.81 | -0.05 | 0.76 | 0.01 | 0.93 | -0.11 | 0.51 |
| **Lutein/Zeaxanthin** | -0.05 | 0.76 | -0.11 | 0.51 | 0.21 | 0.21 | 0.14 | 0.38 | -0.13 | 0.45 |
| **Total Carotenoids** | 0.08 | 0.62 | -0.27 | 0.09 | 0.10 | 0.55 | 0.15 | 0.37 | -0.26 | 0.17 |

$\rho$, Spearman’s correlation coefficients; 7DFR, 7-day food records; BMI, body mass index.

† Supplement use (yes/no).

‡ Smoking status (smoker/non-smoker).
